# *C. elegans* SIRT6/7 Homolog SIR-2.4 Promotes DAF-16 Relocalization and Function during Stress

**DOI:** 10.1371/journal.pgen.1002948

**Published:** 2012-09-13

**Authors:** Wei-Chung Chiang, Daniel X. Tishkoff, Bo Yang, Joshua Wilson-Grady, Xiaokun Yu, Travis Mazer, Mark Eckersdorff, Steven P. Gygi, David B. Lombard, Ao-Lin Hsu

**Affiliations:** 1 Department of Molecular and Integrative Physiology, University of Michigan, Ann Arbor, Michigan, United States of America; 2 Department of Pathology, University of Michigan, Ann Arbor, Michigan, United States of America; 3 Department of Cell Biology, Harvard Medical School, Boston, Massachusetts, United States of America; 4 Department of Internal Medicine, Division of Geriatric Medicine, University of Michigan, Ann Arbor, Michigan, United States of America; 5 Institute of Gerontology and the Geriatrics Center, University of Michigan, Ann Arbor, Michigan, United States of America; University of California San Francisco, United States of America

## Abstract

FoxO transcription factors and sirtuin family deacetylases regulate diverse biological processes, including stress responses and longevity. Here we show that the *Caenorhabditis elegans* sirtuin SIR-2.4—homolog of mammalian SIRT6 and SIRT7 proteins—promotes DAF-16–dependent transcription and stress-induced DAF-16 nuclear localization. SIR-2.4 is required for resistance to multiple stressors: heat shock, oxidative insult, and proteotoxicity. By contrast, SIR-2.4 is largely dispensable for DAF-16 nuclear localization and function in response to reduced insulin/IGF-1-like signaling. Although acetylation is known to regulate localization and activity of mammalian FoxO proteins, this modification has not been previously described on DAF-16. We find that DAF-16 is hyperacetylated in *sir-2.4* mutants. Conversely, DAF-16 is acetylated by the acetyltransferase CBP-1, and DAF-16 is hypoacetylated and constitutively nuclear in response to *cbp-1* inhibition. Surprisingly, a SIR-2.4 catalytic mutant efficiently rescues the DAF-16 localization defect in *sir-2.4* null animals. Acetylation of DAF-16 by CBP-1 *in vitro* is inhibited by either wild-type or mutant SIR-2.4, suggesting that SIR-2.4 regulates DAF-16 acetylation indirectly, by preventing CBP-1-mediated acetylation under stress conditions. Taken together, our results identify SIR-2.4 as a critical regulator of DAF-16 specifically in the context of stress responses. Furthermore, they reveal a novel role for acetylation, modulated by the antagonistic activities of CBP-1 and SIR-2.4, in modulating DAF-16 localization and function.

## Author Summary

Sensing and responding appropriately to environmental insults is a challenge facing all organisms. In the roundworm *C. elegans*, the FoxO protein DAF-16 moves to the nucleus in response to stress, where it regulates gene expression and plays a key role in ensuring organismal survival. In this manuscript, we characterize SIR-2.4 as a novel factor that promotes DAF-16 function during stress. SIR-2.4 is a member of a family of proteins called sirtuins, some of which promote increased lifespan in model organisms. Worms lacking SIR-2.4 show impaired DAF-16 nuclear recruitment, DAF-16–dependent gene expression, and survival in response to a variety of stressors. SIR-2.4 regulates DAF-16 by indirectly affecting levels of a modification called acetylation on DAF-16. Overall, our work has revealed SIR-2.4 to be a key new factor in stress resistance and DAF-16 regulation in *C. elegans*. Future studies will address whether mammalian SIR-2.4 homologs SIRT6 and SIRT7 act similarly towards mammalian FoxO proteins.

## Introduction

Elucidation of mechanisms regulating stress resistance and longevity has been aided tremendously by the use of invertebrate models. FoxO transcription factors regulate multiple biological processes in many organisms [1]. In *C. elegans* and *Drosophila*, increased FoxO activity promotes longevity, fat storage, and stress resistance [2]. Mammals possess four FoxO homologs, with partially redundant and distinct functions: FoxO1, FoxO3A, FoxO4, and FoxO6 [1]. These proteins regulate apoptosis, cell cycle arrest, oxidative defense, DNA repair, metabolism, differentiation, stem cell function, and tumor suppression in a cell type- and context-specific manner [1].

FoxO activity is tightly controlled, and subcellular localization is a principal mechanism of FoxO regulation [3]. In this context, insulin/IGF-1-like signaling (IIS) is the major influence on FoxO function. IIS leads to FoxO phosphorylation and cytoplasmic segregation in a complex with 14-3-3 chaperone proteins. Conversely, stress stimuli promote nuclear translocation of FoxO proteins by multiple mechanisms, including activation of stress kinases that modify FoxO proteins on residues distinct from those phosphorylated in IIS [2]. In response to oxidative insult, mammalian FoxO proteins are acetylated [4], [5], [6], [7], mono-ubiquitylated [8], and phosphorylated [9], [10], [11], [12]. Overall, these post-translation modifications function as a “FoxO code”, providing a means by which FoxO activity is finely regulated in response to various stimuli to promote altered metabolism, stress responses, or cell death [3].

The sirtuins are an evolutionarily conserved protein family impacting many biological processes, including longevity, stress responses, metabolism, and cancer [13]. Sirtuins modify target proteins by means of their NAD^+^-dependent lysine deacetylase and ADP-ribosyltransferase activities. Lysine acetylation has emerged as a post-translational modification with a key role in modulating protein function, akin to phosphorylation [14]. Mammals possess seven sirtuins, SIRT1-SIRT7. The *C. elegans* genome encodes four sirtuins, SIR-2.1 through SIR-2.4, corresponding to mammalian SIRT1 (SIR-2.1), SIRT4 (SIR-2.2 and SIR-2.3), and SIRT6/7 (SIR-2.4) [15]. SIR-2.1 has been implicated in numerous physiologic processes, including stress responses [16], [17], [18], [19], [20], [21], [22], [23]. In contrast, functions of other worm sirtuins are largely uncharacterized [24].

In different organisms, sirtuins modulate FoxO activity via diverse means. In *C. elegans*, there are reports that SIR-2.1 extends longevity in a DAF-16-dependent manner [21], [25], [26], [27]. However, this is currently a disputed finding [28], [29]. In mammals, SIRT1 directly deacetylates FoxO proteins in response to oxidative stress. The effect of FoxO deacetylation is somewhat controversial [4], [6], [7], [30], [31], but it is likely that the overall outcome is to promote DNA repair and cell cycle arrest while inhibiting apoptosis [3]. SIRT2 also deacetylates FoxO proteins to inhibit adipocytic differentiation [32], [33] and regulate levels of intracellular reactive oxygen species (ROS) [34]. SIRT1 and SIRT2-mediated deacetylation of FoxO1 promotes nuclear accumulation of this protein [5], [32]. Acetylation of FoxO1 has also been reported to attenuate DNA binding, to promote AKT-mediated FoxO1 phosphorylation, and to direct FoxO1 to nuclear PML bodies [7], [35]. The mitochondrial sirtuin SIRT3 has also been proposed to modulate FoxO function [36], [37].

Here, we characterize functions of the *C. elegans* sirtuin SIR-2.4, a protein about which little is currently known. We find that SIR-2.4 plays a crucial role in promoting DAF-16 transcriptional activity and stress-induced nuclear localization, and is required for normal stress resistance in the worm. However, SIR-2.4 is largely dispensable for DAF-16 nuclear localization and function in the context of reduced IIS. We show directly for the first time that DAF-16 itself is acetylated, by the acetyltransferase CBP-1. SIR-2.4 attenuates DAF-16 acetylation in a non-catalytic activity-dependent manner, and catalytic function of SIR-2.4 is dispensable for regulation of DAF-16 localization in response to stress. Acetylation of DAF-16 by CBP-1 is inhibited in the presence of SIR-2.4. Our results indicate acetylation plays a key role in regulating DAF-16 localization and function, and that levels of this modification are modulated by the antagonistic functions of CBP-1 and SIR-2.4.

## Results

### SIR-2.4 is required for efficient stress-induced DAF-16 nuclear localization

**Figure 1:**
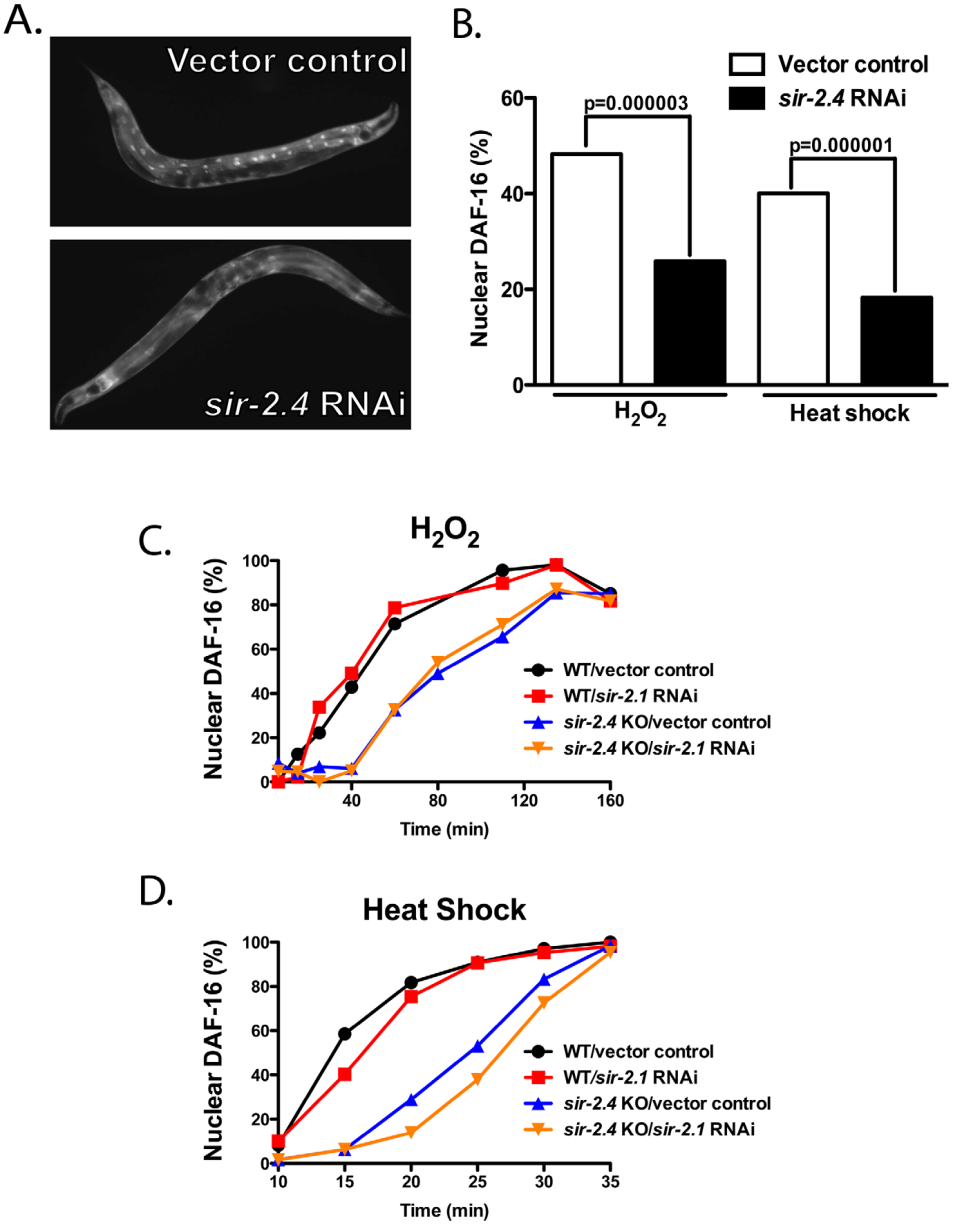
SIR-2.4, but not SIR-2.1, is required for stress-induced DAF-16 nuclear localization. TJ356 animals carrying an integrated *daf-16::gfp* array were fed either vector control or *sir-2.4* RNAi bacteria for at least one generation before being subjected to heat-shock or oxidative stress. (A) Images of TJ356 animals grown on control or *sir-2.4* RNAi bacteria after 15 min heat-shock. (B) Quantification of DAF-16::GFP nuclear accumulation in response to heat-shock (35°C for 15 min.) or oxidative stress (1.5 mM H_2_O_2_ for 1 hr). Worms were scored for the presence or absence of GFP accumulation within the intestinal nuclei (n = 120 or greater for all treatments). An animal was scored as having nuclear GFP if one or more intestinal nuclei contained DAF-16-GFP. (C–D) Time course analysis of DAF-16::GFP nuclear accumulation in response to stress. TJ356 or EQ200 [*sir-2.4(n5137)*; *daf-16::gfp*] animals grown on either control or *sir-2.1* RNAi bacteria were subjected to (C) heat-shock (35°C) or (D) oxidative stress (1.5 mM H_2_O_2_). Worms were scored for GFP accumulation within the head hypodermic nuclei at day 1 of adulthood (n = 30∼50) every 5–30 min.

Mammalian SIRT6 and SIRT7 proteins both promote genotoxic stress resistance [38], [39]. We therefore tested a potential role for SIR-2.4 in stress resistance and DAF-16 regulation. We generated an RNAi construct encoding nucleotides 1–467 of the *SIR-2.4* open reading frame in the RNAi vector L4440. *sir-2.4* knockdown (KD) resulted in no obvious defects under basal conditions. However, *sir-2.4* RNAi severely impaired stress-induced DAF-16 nuclear localization (Figure 1A). *sir-2.4* RNAi inhibited DAF-16 nuclear translocation in response to either heat shock or oxidative insult by ∼50% shortly after stress induction (Figure 1B). At later timepoints, DAF-16 did translocate to the nucleus in *sir-2.4* KD worms (see below and data not shown).

*sir-2.4* deletion is reported to confer lethality/sterility (National Bioresource Project, Japan). However, during the course of analyzing the effects of *sir-2.4* RNAi, we obtained a viable strain with a deletion removing all but the initial 9 amino acids of the *SIR-2.4* open reading frame (kind gift of H.R. Horvitz; see *Materials and Methods* section for a complete description of this strain). As with *sir-2.4* RNAi, *sir-2.4* KO animals showed no apparent defects under unstressed conditions. However, like *sir-2.4* KD worms, *sir-2.4* knockouts (KOs) showed significantly delayed stress-induced DAF-16 nuclear translocation in response to oxidative stress (Figure 1C; p<0.001 by Poisson regression analysis) and heat shock (Figure 1D; p<0.001). We conclude that SIR-2.4 is dispensable for viability and fertility, but plays a crucial role in directing DAF-16 to the nucleus in response to stress, particularly at early time points following stress induction.

It has been reported that SIR-2.1 and 14-3-3 proteins act in concert to activate DAF-16 [21], [26]. To examine whether SIR-2.1 plays a role in directing DAF-16 to the nucleus in response to stress, we assessed the effect of *sir-2.1* KD on DAF-16 nuclear localization. *sir-2.1* KD alone had little impact on DAF-16 nuclear recruitment in response to either oxidative insult (Figure 1C; p<0.72) or heat stress (Figure 1D; p<0.44), indicating that SIR-2.1 does not play a major role in stress-induced DAF-16 nuclear localization, consistent with published data [17]. Moreover, KD of *sir-2.1* in the context of *sir-2.4* mutation did not produce any statistically significant additional delay in DAF-16 nuclear recruitment versus *sir-2.4* KO alone (Figure 1C–1D; p<0.89 and p<0.13 for oxidative and heat stress, respectively). We conclude that SIR-2.4, but not SIR-2.1, plays a major role in promoting rapid nuclear recruitment of DAF-16 in response to oxidative stress or heat shock. These results imply that SIR-2.1 and SIR-2.4 act in distinct pathways to influence DAF-16 functions.

While stress-induced subcellular translocation of DAF-16 was affected in all cell types by *sir-2.4* inhibition, we did note that *sir-2.4* KD and KO had a bigger impact in head hypodermis cells than in intestinal cells (data not shown). SIR-2.4 was very weakly expressed in most cell types, but showed much stronger expression in a subset of head and tail neurons as well as in a subset of somatic gonad cells (Figure S1). We have not yet formally assessed the tissue requirements for SIR-2.4 function in the regulation of DAF-16 localization, though our functional data suggest a cell autonomous mechanism (see below).

### SIR-2.4 promotes DAF-16–dependent transcription under basal and stress conditions

**Figure 2:**
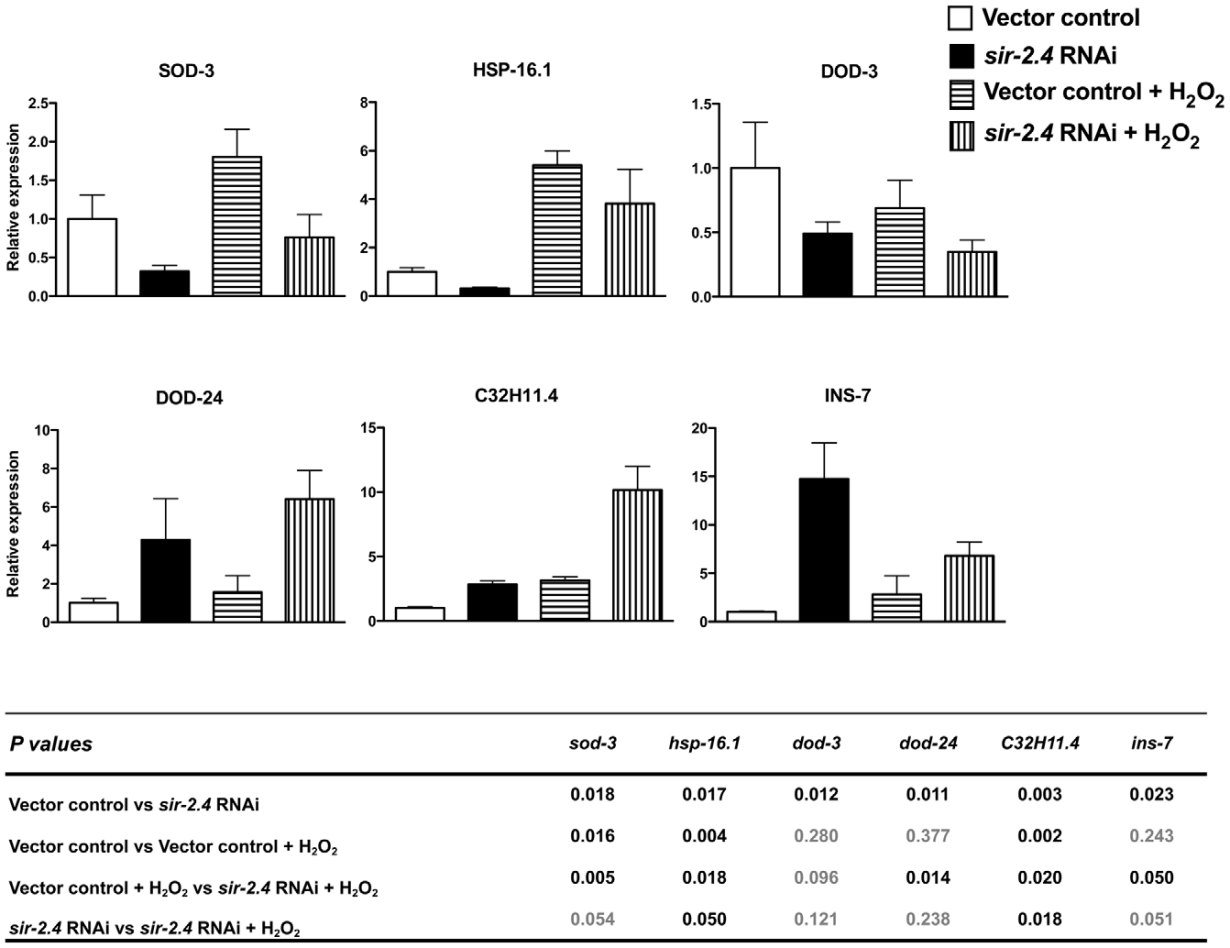
SIR-2.4 is required for optimal DAF-16–dependent gene expression. Wild-type N2 animals fed on either vector control or *sir-2.4* RNAi bacteria from the time of hatching were exposed to 10 mM H_2_O_2_ for 80 min. Relative mRNA levels of SOD-3, HSP-16.1, DOD-3, DOD-24, C32H11.4, and INS-7 were measured by quantitative RT-PCR and the means of three different sample sets are shown. Relative mRNA levels were normalized against ACT-1 (beta-actin). Error bars: ± STD. Statistical significance as determined by two-tailed t-test is shown in the table below; significant differences are represented in black font.

DAF-16 carries out its functions by transcriptional regulation of a large number of target genes [40], [41], [42]. The role of SIR-2.4 in DAF-16-dependent gene expression was tested in the context of six well-known DAF-16 targets. We confirmed the published role of DAF-16 in regulating expression of all six of these genes (Figure S2A). Under both basal and stress conditions, *sir-2.4* RNAi led to decreased mRNA levels of three genes positively regulated by DAF-16 (*SOD-3*, *HSP-16.1*, and *DOD-3*), (Figure 2, top row). Expression of these DAF-16 targets was similarly attenuated in the *sir-2.4* KO strain (Figure S2B). Conversely, expression of three genes negatively regulated by DAF-16 was greatly increased by *sir-2.4* RNAi (*DOD-24*, *C32H11.4*, and *INS-7*) (Figure 2, bottom row). We conclude that SIR-2.4 promotes DAF-16 transcriptional function under both basal and oxidative stress conditions.

### SIR-2.4 is required for normal stress resistance

**Figure 3:**
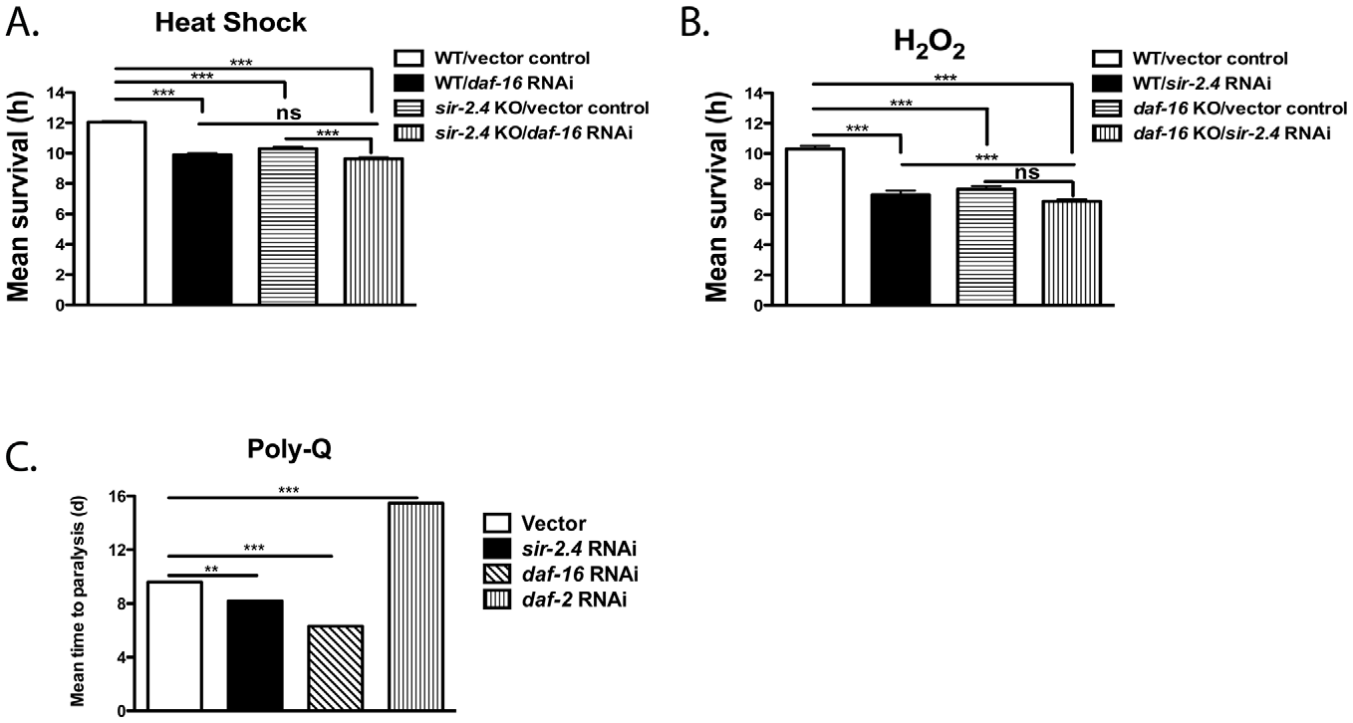
SIR-2.4 promotes stress resistance. (A) Wild-type N2 or *sir-2.4(n5137)* worms grown on vector control or *daf-16* RNAi bacteria were subjected to heat-shock at 35°C and scored for viability every 1-2 hours. (B) Wild-type N2 or *daf-16(mu86)* worms grown on vector control or *sir-2.4* RNAi bacteria were treated with 1.5 mM H_2_O_2_ and scored for viability every 1–2 hours. Data are mean survival±SEM, in hours for (A–B). ***, *p*<0.001; ns, *p*>0.05. See Table S1 for statistical analysis. (C) AM140 worms expressing a poly-Q tract (Q35::YFP) were seeded on the RNAi bacteria indicated and scored for poly-Q induced paralysis every other day. Data are mean time to paralysis in days±SEM. ***, *p*<0.001; **, *p*<0.01; ns, *p*>0.05.

DAF-16 is a key regulator of stress responses in *C. elegans* [43]. We therefore tested the impact of SIR-2.4 on stress resistance. *sir-2.4* KO and *sir-2.4* KD worms were hypersensitive to heat shock (Figure 3A, Table S1) and oxidative insult (Figure 3B, Table S1). Simultaneous inhibition of both *sir-2.4* and *daf-16* increased stress sensitivity to a similar extent as observed in *daf-16* single mutants, and the degree of hypersensitivity conferred by either single KO/KD alone was similar (Figure 3A–3B, Figure S2C–S2D), suggesting that SIR-2.4 and DAF-16 modulate stress resistance via a common pathway. Conversely, overexpression of SIR-2.4 did not produce increased stress resistance (Figure S3A–S3B; Table S1); hence SIR-2.4 levels are not limiting for DAF-16 regulation and stress resistance.

Expression of fluorescently tagged polyglutamine (polyQ) repeat-containing proteins in *C. elegans* body wall muscle causes paralysis that is antagonized by DAF-16 [44], [45], a model for proteotoxicity occurring in human neurodegenerative diseases such as Huntington's disease. The role of SIR-2.4 in proteotoxicity resistance was assessed. Worms expressing 35 glutamine residues conjugated to YFP in body wall muscle (*unc-54p::Q35::YFP*) were grown on *daf-*2 RNAi, *daf-16* RNAi, *sir-2.4* RNAi, or control bacteria. *daf-2* RNAi delayed, and both *daf-16* and *sir-2.4* RNAi accelerated, the onset of Q35::YFP-induced paralysis (Figure 3C). Thus, like DAF-16, SIR-2.4 is required for resistance to multiple stressors, including proteotoxic injury.

### SIR-2.4 is largely dispensable for DAF-16 function in response to reduced IIS

**Figure 4:**
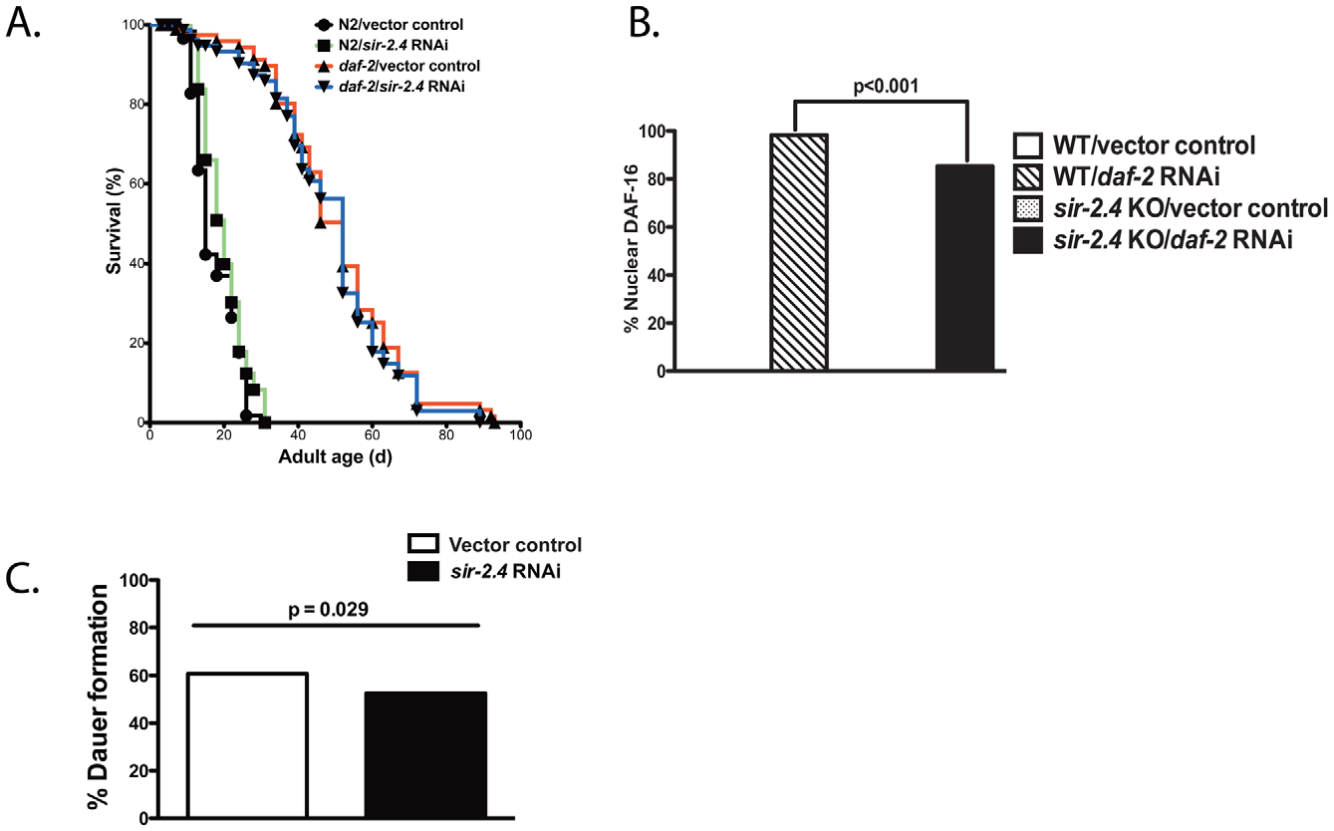
Minimal impact of SIR-2.4 on IIS-induced longevity, DAF-16 nuclear localization induced by reduced IIS, and dauer formation. (A) Lifespan analysis of wild-type (N2) animals or *daf-2(e1370)* mutants grown on vector control bacteria (black or red) or *sir-2.4* RNAi bacteria (green or blue) at 20°C. (B) DAF-16 nuclear localization was assessed in TJ356 (expressing *daf-16::gfp* in WT background) or EQ200 (expressing *daf-16::gfp* in *sir-2.4(n5137)* background) animals. Animals were fed with either vector control or *daf-2* RNAi from the time of hatching. Worms were scored for the presence or absence of GFP accumulation within the head hypodermic nuclei as day 1 adult (n = 116 or greater) under unstressed condition. *P*-values were calculated by Pearson's chi-square test. (C) *daf-2(e1370)* mutants (P_0_) were fed with control or *sir-2.4* RNAi bacteria at 20°C. F_1_ eggs were then moved to 22°C for 72 hours prior to being scored for dauer formation (n = 336 or greater). *P*-values were calculated by Pearson's chi-square test.

Reduced IIS promotes DAF-16 nuclear localization and functions independently of exogenous stress. We performed several assays to determine whether SIR-2.4 regulates DAF-16 in the context of IIS. Many manipulations that reduce IIS increase lifespan in *C. elegans*. However, neither *sir-2.4* RNAi nor *SIR-2.4* overexpression affected the lifespan of wild-type worms (Figure 4A and Figure S3C, Table S2), nor did *sir-2.4* RNAi suppress increased longevity of *daf-2 (e1370)* insulin/IGF-I-like receptor mutants (Figure 4A, Table S2). *sir-2.4* deletion or *sir-2.4* RNAi minimally impacted DAF-16 nuclear translocation induced by reduced IIS (Figure 4B and Figure S2E), in contrast to its potent impact on stress-induced DAF-16 relocalization. Moreover, *sir-2.4* RNAi only slightly impaired dauer formation, a process antagonized by IIS (Figure 4C). We conclude that that the effects of SIR-2.4 on DAF-16 are largely independent of IIS, and are most functionally significant in the context of stress.

### SIR-2.4 regulates DAF-16 acetylation and localization independently of its catalytic function

**Figure 5:**
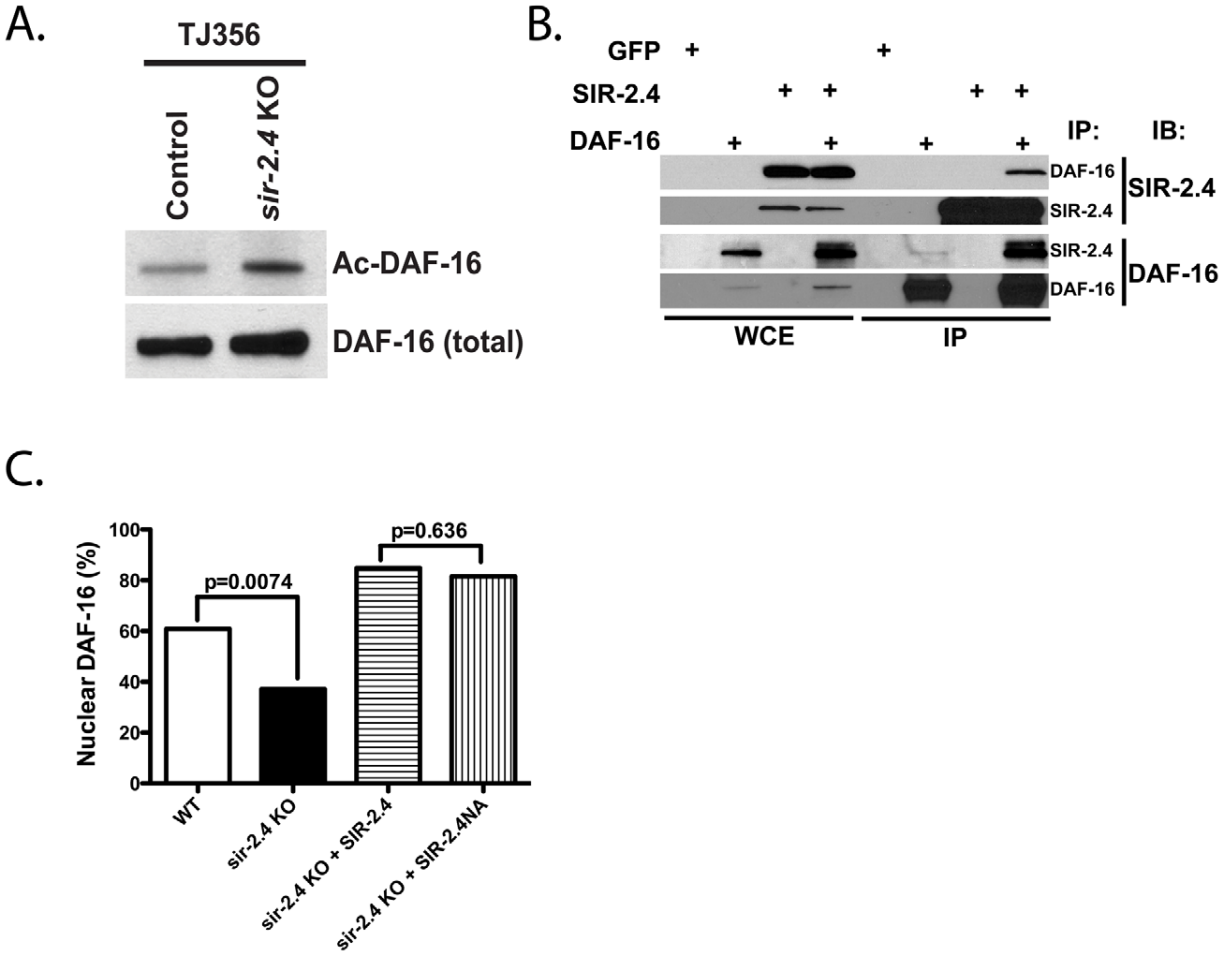
SIR-2.4 interacts with DAF-16 and promotes DAF-16 deacetylation and function independently of catalytic activity. (A) *sir-2.4* deletion promotes DAF-16 hyperacetylation. DAF-16 acetylation was assessed in control or *sir-2.4* KO worms by acetyl-lysine immunoprecipitation followed by GFP immunoblot. (B) SIR-2.4 and DAF-16 interact. Plasmids encoding FLAG-tagged SIR-2.4 and HA-tagged DAF-16 were transfected into 293T cells as indicated (GFP, negative control). Immunoprecipitation and immunoblotting were performed as shown. (C) Rescue of DAF-16 nuclear localization with a catalysis-defective *sir-2.4* mutant. Stable transgenic strains of *sir-2.4(n5137)* were generated expressing either wild-type SIR-2.4 or the *sir-2.4* N124A mutant. Worms were scored for GFP accumulation within the head hypodermic nuclei as day 1 adult (n = 50 or greater) after 20 min of heat-shock at 35°C. *P*-values were calculated by Pearson's chi-square test.

In mammals, SIRT1 and other sirtuins deacetylate FoxO proteins to promote stress responses and other processes [4], [6], [30], [31], [32], [33], [36]. Although DAF-16 interacts with the acetyltransferases CBP and p300 [46], acetylation of DAF-16 itself has never been demonstrated. Given our data linking SIR-2.4 to stress resistance and DAF-16 localization and function, we tested whether DAF-16 was acetylated, and whether SIR-2.4 might play a role in regulating levels of this modification. Indeed, we were able to detect DAF-16 acetylation in *C. elegans*, and levels of this modification were elevated in *sir-2.4* KO (Figure 5A) or KD worms (Figure S4A). Moreover, reciprocal co-immunoprecipitation studies revealed that SIR-2.4 binds to DAF-16 in mammalian cells (Figure 5B), suggesting that SIR-2.4 might interact with DAF-16 to deacetylate this protein. To test this hypothesis, we performed *in vitro* deacetylation assay with purified SIR-2.4 proteins and pre-acetylated DAF-16 as substrates. However, in multiple experiments we were unable to detect significant deacetylation of DAF-16 by SIR-2.4 *in vitro*, despite efficiently deacetylating histones with mammalian SIRT1 and SIRT6 in parallel under identical conditions (data not shown).

Given this result, the requirement for SIR-2.4 catalytic function in DAF-16 localization was directly tested in worms. As shown above, *sir-2.4* KO worms showed impaired DAF-16 nuclear localization in response to stress. This defect was rescued by transgenic expression of wild-type SIR-2.4 (Figure 5C). Curiously, expression of the SIR-2.4 N124A mutant, bearing a mutation at highly conserved residues predicted based on homology to greatly diminish catalytic function [47], restored DAF-16 relocalization as efficiently as wild-type SIR-2.4. Unfortunately, co-overexpression of *DAF-16* together with chromosomally integrated *SIR-2.4* caused worms to be very sick and produce few progeny, precluding a direct assessment of DAF-16 acetylation in rescued animals (data not shown). We conclude that SIR-2.4 regulates DAF-16 acetylation and localization independently of its catalytic activity, potentially by preventing the action of an acetyltransferase on DAF-16 and/or recruiting another deacetylase.

Potential cooperativity between SIR-2.1 and SIR-2.4 with respect to regulation of DAF-16 acetylation was assessed (Figure S4B). As before, *sir-2.4* KO caused a large increase in DAF-16 acetylation. Interestingly *sir-2.1* RNAi caused a modest but detectable increase in DAF-16 acetylation. In *sir-2.1* KD;*sir-2.4* KO animals, levels of DAF-16 acetylation resembled those in the *sir-2.4* KO. Thus, SIR-2.1 does impact DAF-16 acetylation, perhaps by directly deacetylating DAF-16, as has been shown for its homolog mammalian SIRT1. However, in contrast to SIR-2.4, the impact of SIR-2.1 on DAF-16 acetylation is quantitatively insufficient to promote significant DAF-16 nuclear localization (our results and [17]). Alternatively, it is possible that SIR-2.1 and SIR-2.4 modulate acetylation on different DAF-16 lysine residue that regulate divergent aspects of DAF-16 function.

### SIR-2.4 blocks CBP1-dependent DAF-16 acetylation

**Figure 6:**
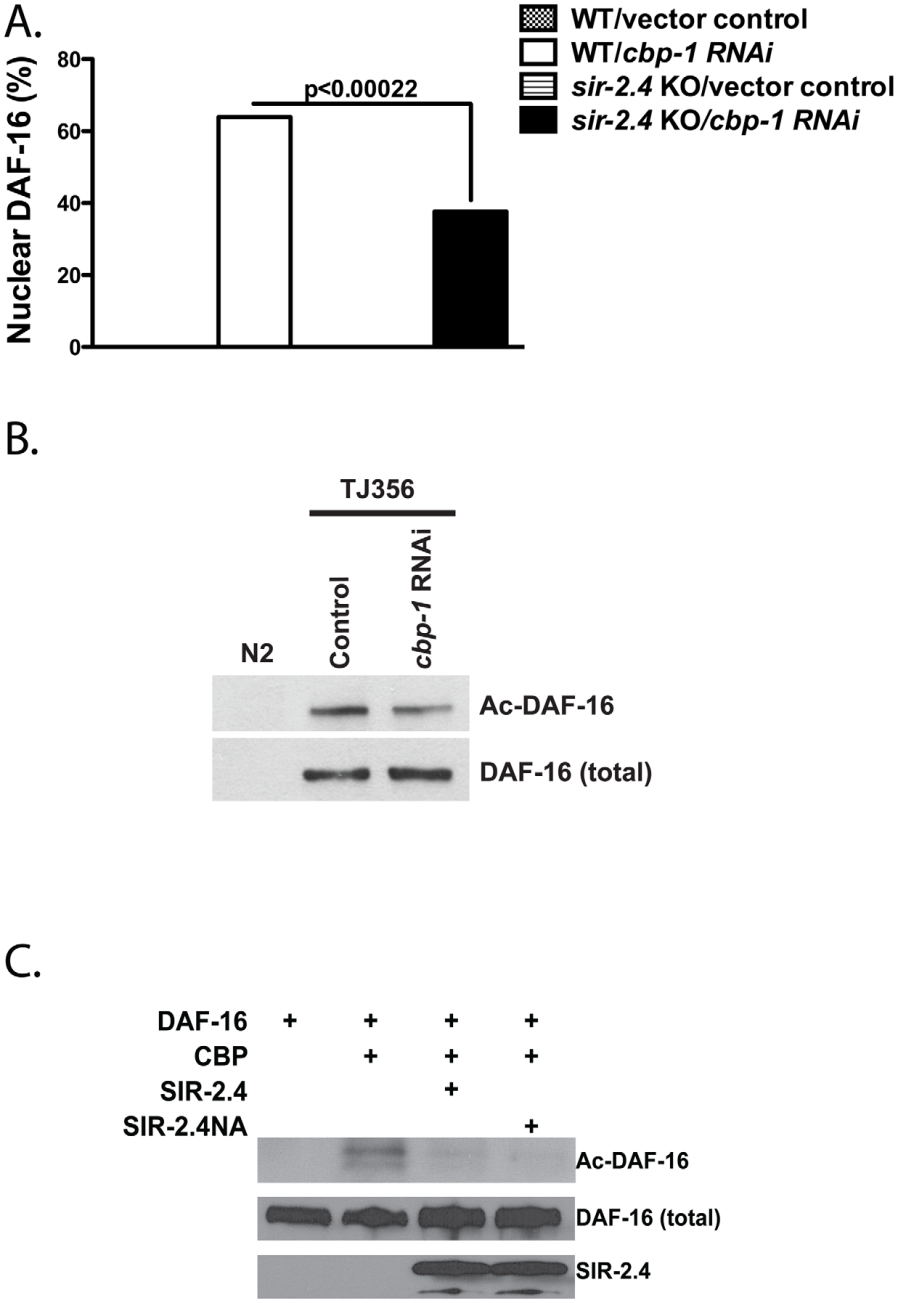
SIR-2.4 inhibits CBP1-mediated DAF-16 acetylation. (A) DAF-16 nuclear localization was assessed in TJ356 (expressing *daf-16::gfp* in WT background) or EQ200 (expressing *daf-16::gfp* in *sir-2.4(n5137)* background) animals. Animals (n = 90 or greater) were scored for DAF-16::GFP nuclear translocation as described in Figure 4B. (B) *cbp-1* KD decreases DAF-16 acetylation. DAF-16 acetylation was assessed in TJ356 worms grown on either control or *cbp-1* RNAi by acetyl-lysine immunoprecipitation followed by GFP immunoblot. (C) SIR-2.4 blocks CBP1-dependent DAF-16 acetylation *in vitro*. Purified DAF-16 was incubated with CBP in the presence of WT SIR-2.4 or the SIR-2.4 NA mutant at 37°C. DAF-16 acetylation levels were assessed as described in (B).

Acetylation regulates of FoxO proteins in a context-specific manner [3]. To identify the enzyme that acetylates DAF-16 *in vivo*, five *C. elegans* acetyltransferases – *CBP-1* (p300/CBP), *PCAF-1* (p300/CBP-associated factor), *MYS-1*, *MYS-2* and *MYS-3* (MYST histone acetyltransferases) – were examined for potential impacts on nuclear translocation and acetylation of DAF-16. RNAi KD of *cbp-1* induced dramatic, virtually complete DAF-16 nuclear translocation under basal, unstressed conditions (Figure 6A). In contrast, inhibition of *pcaf*-1 or simultaneous KD of all three MYST acetyltransferases had no effect on DAF-16 localization (data not shown). This effect of *cbp-1* RNAi on DAF-16 nuclear localization was largely epistatic to the impact of *sir-2.4* KO (Figure 6A). These results are consistent with a recent report showing that mammalian CBP promotes cytoplasmic localization of FoxO3A [48]. Consistent with a role for CBP-1 in acetylating DAF-16 *in vivo*, we found that DAF-16 is hypo-acetylated in *cbp-1* KD animals (Figure 6B). To identify CBP-1 target sites on DAF-16, we performed mass spectrometry analysis on recombinant DAF-16 treated with CBP-1 *in vitro* (Figure 6C). We identified four CBP-1 dependent acetylation sites on DAF-16: K248, K253, K375 and K379 (Table S3). Together, these findings suggest that DAF-16 is acetylated by CBP-1 *in vivo* to promote its nuclear exclusion under basal conditions.

It has been reported that *cbp-1* RNAi confers stress sensitivity and reduced lifespan [49]. We confirmed that *cbp-1* RNAi caused hypersensitivity to heat shock (Figure S5A) and oxidative insult (Figure S5B). Moreover, as previously reported, *cbp-1* RNAi caused worms to be very short-lived (Figure S5C). We conclude that the constitutive nuclear localization of DAF-16 occurring in the context of *cbp-1* inhibition is inadequate to promote stress resistance or increased longevity, perhaps due to loss of *cbp-1* acetylation of other key targets necessary for health and normal lifespan, potentially in many different cell types. Consistent with this hypothesis, CBP-1 is widely express in most or all somatic cells of the worms [50], [51], [52].

**Figure 7:**
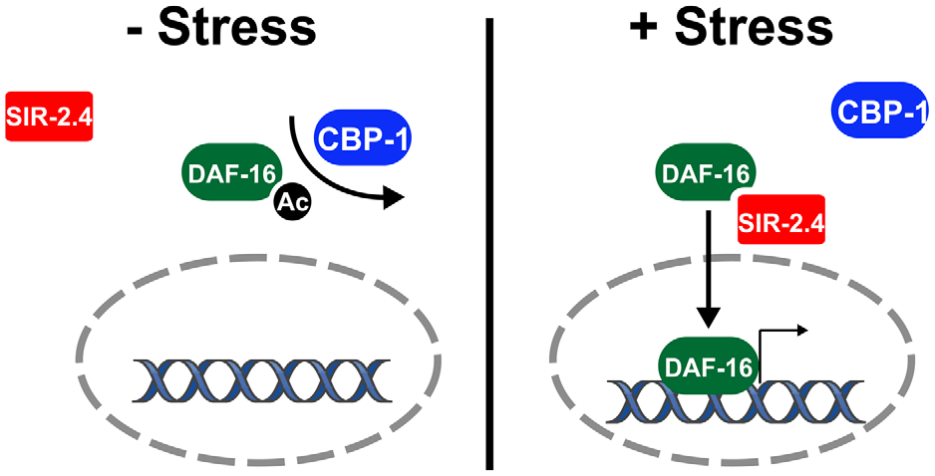
Model: SIR-2.4 promotes DAF-16 deacetylation and function during stress. See text for details.

The DEK protein binds histones to prevent p300- and PCAF-mediated histone acetylation [53]. To test whether SIR-2.4 might exert its effect on DAF-16 through a similar mechanism, we examined the effect of the presence of SIR-2.4 on CBP-mediated DAF-16 acetylation *in vitro*. Purified DAF-16 and CBP were incubated with or without SIR-2.4. The presence of SIR-2.4 blocked CBP-mediated acetylation of DAF-16 (Figure 6C). A similar result was also obtained when DAF-16 and CBP were incubated with the SIR-2.4 N124A catalytic mutant (Figure 6C). We conclude that SIR-2.4 inhibits CBP-mediated DAF-16 acetylation likely through protein-protein interaction, independent of deacetylase function (Figure 7).

## Discussion

We have shown that the sirtuin SIR-2.4 plays a crucial role in promoting DAF-16 activity and stress resistance. In response to multiple forms of stress, SIR-2.4 promotes DAF-16 nuclear recruitment and function, and ultimately organismal survival. SIR-2.4 is also required for optimal DAF-16 deacetylation and transcriptional activity under basal, unstressed conditions. In contrast, SIR-2.4 does not greatly impact DAF-16 nuclear localization, dauer formation, or longevity due to reduced IIS. We find that SIR-2.4 associates with DAF-16 and negatively regulates DAF-16 acetylation by interfering with the ability of CBP-1 to acetylate DAF-16 under stress conditions. Although mammalian FoxO proteins are acetylated in a functionally significant manner [3], to our knowledge this is the first demonstration that this modification is conserved in *C. elegans*. These data suggest that deacetylation likely plays two distinct roles in modulating DAF-16 function, in promoting optimal DAF-16 transcriptional activity under both basal and stress conditions, and in promoting DAF-16 nuclear recruitment following stress. We propose a model in which SIR-2.4 does not interact with DAF-16 under unstressed conditions. This allows CBP-1 to acetylate DAF-16 and sequester DAF-16 in the cytosol. In response to stress, SIR-2.4 binds to DAF-16 to block CBP-1-depedent acetylation and promote DAF-16 nuclear translocation and gene expression (Figure 7).

There are several important issues raised by our results. First, our data suggest that deacetylation promotes DAF-16 nuclear localization and function in response to stress, but is largely dispensable in the context of reduced IIS, suggesting that regulation of this modification may be involved in adjusting DAF-16 activity in the face of different organismal requirements. Our mass spectrometry analysis has identified four acetylation sites on DAF-16 (Table S3). Notably, acetylation at one of these (K248), is also present at homologous lysine residues in FoxO1 (K262) and FoxO3A (K259) [4], [54], [55], [56]. Future studies mutating these acetylation sites individually and in combinations will be required to identify specific residues involved in regulating DAF-16 nuclear recruitment and transcriptional activity.

Our results provide further evidence that multiple sirtuins regulate FoxO proteins by diverse mechanisms to achieve different functional outcomes. In mammals, several sirtuins directly deacetylate FoxO proteins [4], [6], [30], [31], [32], [33], [36]. In worms, SIR-2.1 promotes DAF-16 function to increase stress resistance via 14-3-3 proteins [26]. We find SIR-2.4 suppresses DAF-16 acetylation and is critical in the organismal response to a variety of stressors. Whereas SIR-2.4 is required for optimal DAF-16 nuclear recruitment in response to stress, SIR-2.1 is dispensable for this process (Figure 1C-1D) [17]. It has been reported that SIRT1 promotes survival of cerebellar granular neurons in response to low extracellular potassium levels in a catalytic activity-independent fashion [57]. Thus, sirtuins may regulate stress responses through mechanisms other than direct covalent substrate modification. Consistent with this idea, we found that SIR-2.4 regulates DAF-16 function independent of its catalytic function, by preventing CBP-1-mediated DAF-16 acetylation. We note that SIRT6 was recently shown to be capable of binding NAD^+^ and undergoing a conformational change in the absence of substrate; based on this finding, it has been speculated that SIRT6 might act as an NAD^+^ metabolite sensor, independently of catalytic activity [58]. This hypothesis is consistent with our data regarding the SIRT6 homolog SIR-2.4. Chromatin modifying complexes frequently involve multiple enzymes with apparently redundant biochemical activities, including multiple histone deacetylases; analogously SIR-2.4 likely associate with other deacetylases to regulate DAF-16. SIR-2.1 associates with DAF-16, although SIR-2.1 was not shown to deacetylate DAF-16 [21], [26]. DAF-16 deacetylases could in principle be SIR-2.1 or other worm sirtuins, or non-sirtuin deacetylases. However, the fact that SIR-2.1 does not play a major role in stress-induced DAF-16 nuclear recruitment strongly suggests that SIR-2.1 is not likely the major DAF-16 deacetylase.

FoXO proteins has been shown to interact with acetyltransferases CBP and p300 in mammalian cells [46], although it was previously unknown whether these proteins directly acetylate DAF-16 in worms. In this study, we showed that inactivation of *cbp-1* by RNAi results in constitutive nuclear localization of DAF-16, and that CBP-1 can directly acetylate DAF-16 on multiple residues *in vitro* (Figure 6C, Table S3). However, *cbp-1* KD only modestly reduces the level of DAF-16 acetylation *in vivo* (Figure 6B). Since the pan-acetyl-lysine antibody we used in these experiments is not able to discriminate among DAF-16 species acetylated at different sites, or singly versus multiply acetylated DAF-16 species, it is possible that CBP-1 is not the only acetyltransferase that acetylates DAF-16 *in vivo*. Thus the effect of *cbp-1* KD on DAF-16 acetylation may be partially obscured by CBP-1-independent acetylation of DAF-16. Nevertheless, CBP-1 has a major impact on DAF-16 localization (Figure 6A).

Finally, it is currently unclear whether SIR-2.4 regulates DAF-16 in a cell-autonomous matter, or whether SIR-2.4 is required in only a subset of cells to carry out this function. The tissue expression pattern of endogenous SIR-2.4 is ambiguous. A SIR-2.4::GFP translational fusion protein shows weak expression in most cell types, but much stronger expression in a subset of head and tail neurons as well as in a subset of somatic gonad cells (Figure S1). Thus, SIR-2.4 could conceivably regulate DAF-16 in neurons to influence the systemic hormonal milieu – *e.g.*, by reducing INS-7 expression – thereby impacting DAF-16 localization and function throughout the animal. However, our observation that DAF-16 is hyperacetylated in extracts from whole worms, together with our biochemical data, supports the hypothesis that SIR-2.4 regulates DAF-16 acetylation in most tissues of the animal cell autonomously. Overall, SIR-2.4 is a novel regulator of DAF-16 localization and function in response to stress in *C. elegans*.

## Materials and Methods

### Strains

CF1041: *daf-2(e1370)*III, TJ356: *zIs356* [*daf-16::gfp* + *rol-6*], AM140: *rmIs132*[*unc-54p*::*Q35::yfp*], MT18068: *sir-2.4(n5137)*I, EQ 137: *iqEx*47 [*sir-2.4p::sir-2.4::gfp* + *rol-6*], EQ158: *iqEx*50 [*sir-2.4p::sir-2.4* + *myo-3p::rfp*], EQ 200: *sir-2.4(n5137)*I; *zIs356* [*daf-16::gfp* + *rol-6*], EQ205: *sir-2.4(n5137)*I; *zIs356* [*daf-16::gfp* + *rol-6*]; iqEx59 [*sir-2.4p::sir-2.4NA* + *myo-3p::rfp*], EQ211: *sir-2.4(n5137)*I; *zIs356* [*daf-16::gfp* + *rol-6*]; iqEx60 [*sir-2.4p::sir-2.4* + *myo-3p::rfp*].

All strains used were maintained and handled as described previously [59]. TJ356, AM140 and CF1041 were obtained from the Caenorhabditis Genetic Center. For the generation of transgenic animals, plasmid DNA mixes were injected into the gonad of young adult hermaphrodite animals, using the standard method described previously [60]. F_1_ progeny were selected on the basis of the roller phenotype. Individual F_2_ progenies were isolated to establish independent lines. For the generation of the EQ137 (SIR-2.4::GFP overexpressor) strain, plasmid DNA containing a mixture of 100 ng/ml of *sir-2.4p::sir-2.4::gfp* and 50 ng/ml of pRF4 (*rol-6*) constructs was injected into N2 animals. For the generation of EQ158 (native SIR-2.4 overexpressor), plasmid DNA containing a mixture of 30 ng/ml of native *SIR-2.4* driven by its own promoter and 50 ng/ml of coinjection marker *myo-3p::rfp* was injected into N2 animals. For the generation of EQ211, plasmid DNA containing a mixture of 30 ng/ml of *sir-2.4p::sir-2.4* and 80 ng/ml of *myo-3p::rfp* constructs was injected into TJ356 animals. For the generation of EQ205, plasmid DNA containing a mixture of 30 ng/ml of *sir-2.4p::sir-2.4NA* and 80 ng/ml of *myo-3p::rfp* constructs was injected into TJ356 animals.

### RNA–interference (RNAi) experiments

HT115 bacteria transformed with RNAi vectors (L4440) expressing dsRNA of the genes indicated were grown at 37°C in LB with 10 mg/mL tetracycline and 50 mg/mL carbenicillin, then seeded onto NG-carbenicillin plates and supplemented with 100 µL 0.1 M IPTG. The *sir-2.4* RNAi construct was generated by cloning nucleotides 1–467 of the *SIR-2.4* cDNA into the L4440 vector. The identity of all RNAi clones was verified by sequencing the inserts using M13-forward primer. Eggs were added to plates and transferred to new plates every 3–6 days.

### *sir-2.4* deletion mutant analysis

The deletion in a *sir-2.4* mutant strain (kind gift of the Horvitz laboratory) was mapped by PCR, and found to encompass 1,929 bp of chromosome I (5990775–5992703), which encodes nucleotides 28–879 of the SIR-2.4 spliced mRNA (data available upon request).

### DAF-16 nuclear localization assay

For quantification of DAF-16::GFP localization, synchronized eggs from TJ356 animals (*i.e.* transgenic animals expressing DAF-16::GFP) or other strains as indicated were seeded onto either vector control or appropriate RNAi plates. For stress response experiments, day 1 adults were washed with M9 three times and transferred to new plates or and subjected to heat shock (35°C) or oxidative stress (1.5 mM H_2_O_2_ in M9). GFP localization was then analyzed using an Olympus BX61 fluorescent microscope at 40× or 100× magnification. For time-course analysis, worms were scored for the presence or absence of GFP accumulation within the nuclei of head hypodermis cells (n = 30∼50) in a blinded fashion every 5–30 min. An animal was scored as having nuclear GFP if more than one head hypodermic nuclei contained DAF-16::GFP. For single time point experiments, worms were blindly scored for the presence or absence of GFP accumulation within the nuclei of indicated cells (n = 120 or greater). *P* values were calculated by Poisson regression (time-course assays) or chi-square test (single time point assays).

### Quantitative RT–PCR analysis

Total RNA was isolated from approximately 5,000 day 1 adult worms, and cDNA was generated from 4 µg of RNA using Superscript III RT (Invitrogen). Real-time qRT-PCR experiments were performed using the Power SYBR Green PCR Master mix (Applied Biosystems) and the Chromo 4 system (MJ Research). Relative mRNA level of the genes of interest were calculated and normalized against an internal control (ACT-1; worm β-actin). Primer sequences were (all 5′-3′): SOD-3 (GTTTCAGCGCGACTTCGGTTCCCT, CGTGCTCCCAAACGTCAATTCCAA); DOD-3 (AAAAAGCCATGTTCCCGAAT, GCTGCGAAAAGCAAGAAAAT); DOD-24 (TGTCCAACACAACCTGCATT, TGTGTCCCGAGTAACAACCA); C32H11.4 (TTACTTCCCATCGCCAAAGT, CAATTCCGGCGATGTATGAT); HSP-16.1 (GATCAAAAGTTTGCCATAAATCTC, TTCAGTCTTTAATTCTTGTTCTCC); INS-7 (TCGTTGTGGAAGAAGAATACATTC, TTAAGGACAGCACTGTTTTCG); and ACT-1 (CTACGAACTTCCTGACGGACAAG, CCGGCGGACTCCATACC).

### Stress assays

For thermotolerance assays, eggs from N2 worms were transferred to plates seeded with vector control, *daf-16* RNAi, or *sir-2.4* RNAi bacteria and grown to day 1 of adulthood. Worms were then transferred to plates without any food and heat-shocked at 35°C. Viability was determined at the indicated timepoints; death was determined by the lack of movement after prodding. For oxidative stress assays, eggs from N2 worms were transferred to plates seeded with vector control, *daf-16* RNAi, or *sir-2.4* RNAi bacteria and grown to day 3 of adulthood. Worms were then transferred to 24-well plates and soaked in 1.5 mM H_2_O_2_ in M9 media. Viability was determined at the indicated timepoints as above. For stress assays, a Kaplan–Meier survival analysis with a log-rank test was performed, and a *P*-value of 0.05 was considered statistically significant.

### Paralysis assay

Synchronized eggs from AM140 animals (i.e. transgenic animals expressing Q35::YFP) were seeded on either vector control or the indicated RNAi bacteria. Animals were scored for polyQ-induced paralysis every other day during adulthood. Paralyzed worms were identified as those failing to make forward or backward movement in response to stimulation by plate-tapping and tail-prodding; these worms still exhibited pharyngeal pumping. For the paralysis assay, a Kaplan–Meier survival analysis with a log-rank test was performed.

### Lifespan analysis

Lifespan analysis were conducted at 20°C as described previously [61], [62]. Strains were grown at 20°C for at least two generations without starvation prior to lifespan analysis. At least 60 worms were used for each experiment. In all experiments, the pre-fertile period of adulthood was used as t = 0 for lifespan analysis. Stata 8 software was used for statistical analysis to determine the means and percentiles. In all cases, *P*-values were calculated using the log-rank (Mantel-Cox) method.

### Assessment of DAF-16 acetylation in worms

∼15,000 synchronized day 1 adult worms grown at 20°C were harvested by washing three times with cold M9 buffer and once with HB-high salt buffer (10 mM HEPES, pH 7.9; 10 mM KCl; 1.5 mM MgCl_2_; 0.1 mM EDTA; 0.5 mM EGTA; 44 mM Sucrose; 100 mM NaCl; 0.5% Triton X-100). Worm pellets were then resuspended in 3× volume of HB-high salt buffer supplemented with Protease Inhibitor Cocktail Complete (Roche), 20 mM β-glycerophosphate, 1 mM sodium orthovanadate, 1 mM nicotinamide and 1 µM trichostatin A. Pellets were immediately frozen and stored in liquid nitrogen. Frozen suspensions were thawed, homogenized with a Dounce homogenizer (30 strokes with pestle B), and centrifuged at 14,000×g at 4°C for 20 minutes. Supernatants were collected and total protein concentrations were quantified by Bradford assay. For immunoprecipitation, 30 µl of anti-acetyl-lysine agarose beads (Immunechem; ICP0388) were added to 1 mg of protein extract with 100 µg/ml of ethidium bromide and incubated with gentle shaking at 4°C overnight. The beads were then washed 3 times with HB-high salt buffer supplemented with 50 mg/ml ABESF, 1 mM sodium orthovanadate, 1 mM nicotinamide and 1 µM trichostatin A. before being subjected to western blotting analysis. The samples were subjected to SDS-PAGE and transferred to a PVDF membrane (Millipore). The membrane was washed three times with TBS containing 0.1% Tween 20 (TBST). After blocking with TBST containing 5% nonfat milk for 60 min, the membrane was incubated with the primary antibody indicated (e.g. anti-GFP, Abcam, #AB6556) at 4°C for 12 h and washed three times with TBST. The membrane was then probed with HRP-conjugated secondary antibody for 1 h at room temperature and washed with TBST three times. Finally, the immunoblots were developed using a chemiluminescent substrate (Millipore) and visualized by autoradiography.

### Assessment of SIR-2.4/DAF-16 interaction

10 cm dishes of HEK293T cells were transfected using TransIT-293 (Mirus) with plasmids encoding HA-tagged DAF-16 (1 µg), FLAG-tagged SIR-2.4 (9 µg), both DAF-16 and SIR-2.4 plasmids together, or a plasmid encoding GFP (5 µg), to assess transfection efficiency. Cells were harvested 48 hours post transfection, and were lysed by rotation at 4°C for 20 minutes in lysis buffer (LB; 150 mM NaCl, 1% Triton X-100, 0.5% NP40, 50 mM Tris pH 7.4, 10% glycerol, with Protease Inhibitor Cocktail Complete-EDTA free (Roche) [63], followed by brief sonication. 1 mg of whole cell extract from each cell line was pre-cleared by slow rotation with 50 µl of protein G conjugated agarose beads. For immunoprecipitation, either 25 µl of M2-agarose beads (Sigma) (anti-FLAG IP) or 25 µl of anti-HA-agarose beads (Roche) (anti-HA IP) was added to pre-cleared WCE, and IPs were incubated overnight by slow rotation at 4°C. After incubation, beads were pelleted and washed three times in LB. FLAG-tagged proteins were eluted in 80 µl of FLAG elution buffer (150 ng/µl 3×FLAG peptide (Sigma), 10 mM Tris-HCl (pH 8.0) and 150 mM NaCl) at 4°C for 4 hours. 40 µl of eluate was loaded on an SDS-PAGE gel. For HA IPs, 80 µl of Laemmli buffer was added to the anti-HA-agarose beads, and beads were boiled at 100°C for 5 minutes; 40 µl was loaded on a gel. DAF16 and SIR2.4 interaction was assessed by immunoblot using anti-DAF-16 (Santa Cruz) or anti-HA antibodies.

### *In vitro* DAF-16 acetylation assay

3×FLAG tagged DAF-16, CBP or SIR-2.4 were each transfected and expressed in 293T cells. These proteins were then purified with Anti-FLAG M2 Affinity Gel (Sigma, A2220). 0.5 µg of purified DAF-16 was incubated with 0.2 µg of purified CBP with or without 0.5 µg of purified SIR-2.4 in the presence of HAT buffer (50 mM Tris-HCl, pH 8.0, 0.1 mM EDTA, 1 mM dithiothreitol, 10%glycerol) supplemented with 200 µM of acetyl-CoA. The reactions were incubated at 37°C for 3 hours and analyzed by western blot. Acetylated DAF-16 was queried with anti-acetyl-lysine antibody (Immunechem; ICP0380) and total level of DAF-16 were assessed with anti-FLAG antibody (Sigma, #F3165).

## Supporting Information

Figure S1 Expression pattern of *sir-2.4* in *C. elegans*. Transgenic lines expressing a SIR-2.4 translational GFP fusion (*sir-2.4p::sir-2.4::gfp*) were utilized to analyze the expression pattern of *sir-2.4* in *C. elegans*. Images of (a–b) L3 or (c–e) late L4/young adult stage transgenic animals (EQ137) expressing GFP protein under control of the *sir-2.4* promoter. *sir-2.4* is highly expressed in a subset of head and tail neurons beginning at early larval stage, indicated by white arrows. High expression of *sir-2.4* is also found in spermathecal-uterine valve (sp-ut valve) cells beginning at L4 larval stage, indicated by white arrows. The yellow arrow indicates the nuclear accumulation of SIR-2.4::GFP fusions in these cells. It is worth noting that very weak expression of *sir-2.4* is found ubiquitously in most tissues, although it is difficult to capture in these images.
(PDF)

Figure S2 Effects of *sir-2.4* RNAi or deletion on gene expression, stress resistance, and IIS-mediated DAF-16 translocation. (A–B) Expression of the genes indicated was measured in worms of the indicated genotypes as in Figure 2. (C) Thermotolerance was assessed in worms of the indicated genotypes as in Figure 3A. (D) Oxidative stress resistance was assessed in worms of the indicated genotypes as in Figure 3B. (E) TJ356 animals were fed with either vector control, *daf-2* RNAi, a 1∶1 mix of *daf-2* and *sir-2.4* RNAi, or a 1∶1 mix of *daf-2* and *yk615e* RNAi bacteria from the time of hatching. *yk615e* is a gene randomly selected as a negative control in double RNAi experiments. Animals (n = 125 or greater) were scored for DAF-16::GFP nuclear translocation as described in Figure 4B.
(PDF)

Figure S3 Minimal impact of SIR-2.4 overexpression on stress resistance or lifespan. Transgenic animals overexpressing (A) a SIR-2.4::GFP fusion protein or (B) native untagged SIR-2.4 protein and N2 controls were exposed to 35°C heat stress. Viability was then scored at the timepoints indicated. (C) Survival curves of wild-type (N2) animals or transgenic animals overexpressing native SIR-2.4 (EQ158) at 20°C. All statistical details are presented in Tables S1 and S2.
(PDF)

Figure S4 *sir-2.4* RNAi promotes DAF-16 hyperacetylation. (A) DAF-16 acetylation was assessed in control or *sir-2.4* RNAi worms by acetyl-lysine immunoprecipitation followed by GFP immunoblot as described in Figure 5A. (B) DAF-16 acetylation was assessed in *sir-2.4* KO, *sir-2.1* RNAi, or double loss-of function animals as indicated.
(PDF)

Figure S5 *cbp-1* loss of function confers stress sensitivity and shortened lifespan. Mean survival of *cbp-1* RNAi worms in response to heat shock (A) or peroxide stress (B). (C) Lifespan curves of wildtype or *sir-2.4* KO animals in the presence of *cbp-1* or control RNAi bacteria.
(PDF)

Table S1 Effects of sir-2.4 expression on stress resistance. Animals indicated were exposed to heat or oxidative stress. Mean survival ± SEM, in hours, observed in the stress analysis is shown. 75th percentile is the time at which the fraction of animals alive reaches 0.25. ‘n’ indicates the number of animals scored in the each experiment. *P*-Values calculated by pair-wise comparisons to vector control of the same experiment. We used Stata 8 software for statistical analysis and to determine means and percentiles. The logrank (Mantel-Cox) test was used to test the hypothesis that the survival functions among groups were equal. a *P*-Values calculated by pair-wise comparisons to N2 grown on vector control of the same experiment. b Compared to N2 grown on the same RNAi bacteria. c Compared to the same mutants grown on vector control. ‘*’ indicates the sets of experiments plotted and shown in Figures.
(PDF)

Table S2 Effects of *SIR-2.4* on lifespan. Adult mean lifespan ± SEM, in days, observed in lifespan analyses. Lifespan experiments were carried out at 20°C. 75th percentile is the age at which the fraction of animals alive reaches 0.25. ‘n’ shows the number of observed deaths relative to total number of animals started at day 1. The difference between these numbers represents the number of animals censored during the experiment, and includes animals that exploded, bagged (i.e. exhibited internal progeny hatching), or crawled off the plates. a *p*-Values calculated by pair-wise comparisons to N2 control of the same experiment. b p-Values calculated by pair-wise comparisons to mutants fed with control bacteria of the same experiment. We used Stata 8 software for statistical analysis and to determine means and percentiles. The logrank (Mantel-Cox) test was used to test the hypothesis that the survival functions among groups were equal.
(PDF)

Table S3 Peptides representing unique acetylated sequences from the FOXO transcription factor are shown with additional information. A“#” sign indicates the site of acetylation in the sequence. The site position refers to which lysine residue on FOXO the identified acetylation site represents. The mass to charge ratio (m/z) and charge state of the peptide as they were observed in the mass spectrometer are given. The mass error specifies the difference between the observed m/z ratio and the theoretical m/z ratio of a peptide, reported in part per million (PPM). The XCorr is the cross correlation score between the theoretical and observed MS^2^ spectra for the matched peptide. The unique ΔCorr is the difference between the XCorr of the top ranking peptide match (the reported peptide) and the XCorr of the next closest ranked peptide of a unique primary sequence (not simply an alternate placement of the acetylation site), normalized by the XCorr of the top ranking peptide match. All reported peptides passed a cutoff of a false discovery rate (FDR) <0.1%, based on the target-decoy strategy [61]. See Text S1 for materials and methods used in these studies.
(DOCX)

Text S1 Supplemental materials and methods.
(DOCX)
