# Supplementary figures and images for: C. elegans SIRT6/7 Homolog SIR-2.4 Promotes DAF-16 Relocalization and Function during Stress
Source: PLoS Genet. 2012 Sep 13;8(9):e1002948. doi: 10.1371/journal.pgen.1002948 (PMC3441721; doi:10.1371/journal.pgen.1002948)

**Fig. S1**

**SIR-2.4::GFP**

**L3**

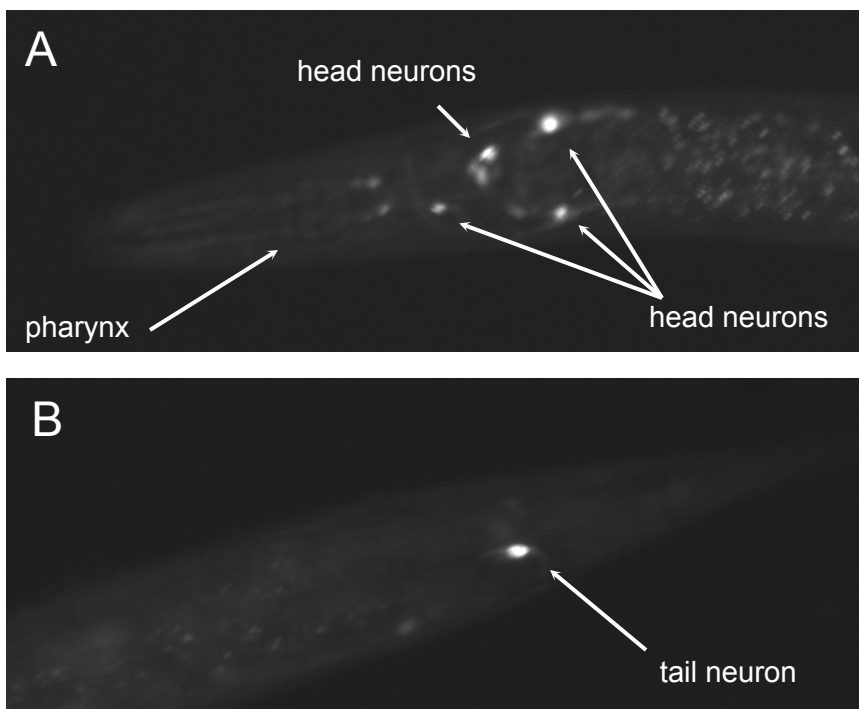

**L4/YA**

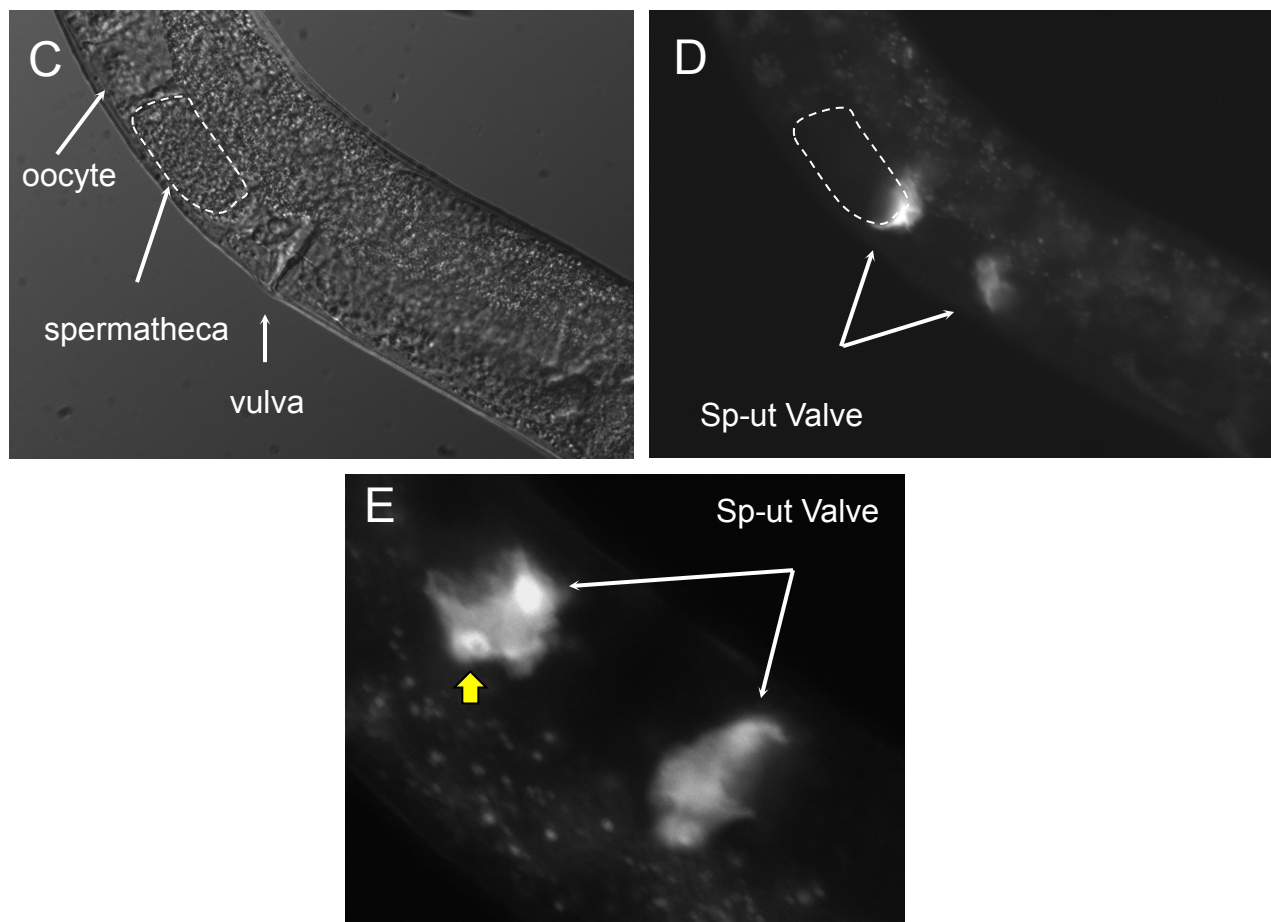

Supplement: Figure S1 — Expression pattern of sir-2.4 in C. elegans. Transgenic lines expressing a SIR-2.4 translational GFP fusion (sir-2.4p::sir-2.4::gfp) were utilized to analyze the expression pattern of sir-2.4 in C. elegans. Images of (a–b) L3 or (c–e) late L4/young adult stage transgenic animals (EQ137) expressing GFP protein under control of the sir-2.4 promoter. sir-2.4 is highly expressed in a subset of head and tail neurons beginning at early larval stage, indicated by white arrows. High expression of sir-2.4 is also found in spermathecal-uterine valve (sp-ut valve) cells beginning at L4 larval stage, indicated by white arrows. The yellow arrow indicates the nuclear accumulation of SIR-2.4::GFP fusions in these cells. It is worth noting that very weak expression of sir-2.4 is found ubiquitously in most tissues, although it is difficult to capture in these images. (PDF) [file pgen.1002948.s001.pdf]

Fig. S2

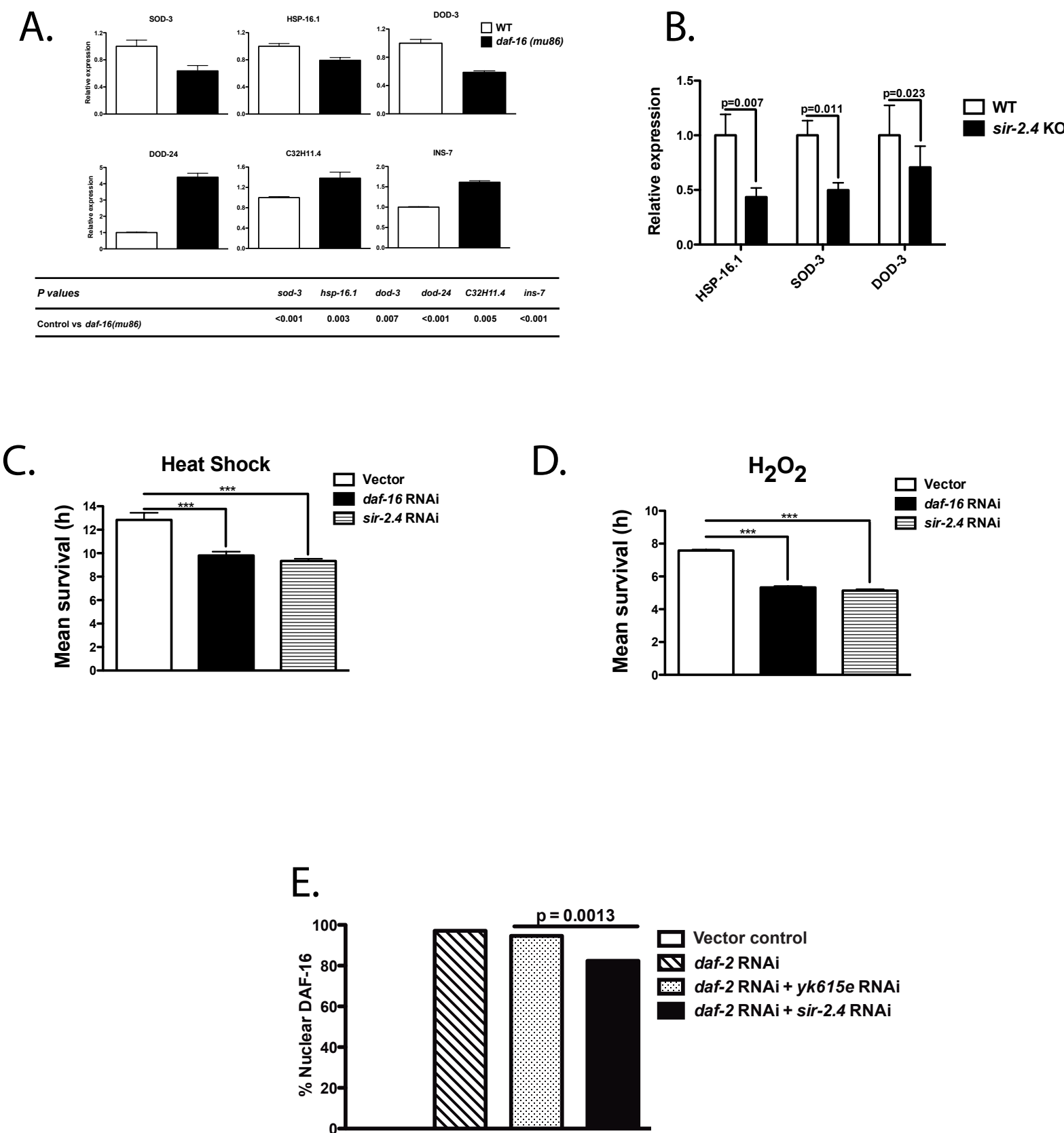

Supplement: Figure S2 — Effects of sir-2.4 RNAi or deletion on gene expression, stress resistance, and IIS-mediated DAF-16 translocation. (A–B) Expression of the genes indicated was measured in worms of the indicated genotypes as in Figure 2. (C) Thermotolerance was assessed in worms of the indicated genotypes as in Figure 3A. (D) Oxidative stress resistance was assessed in worms of the indicated genotypes as in Figure 3B. (E) TJ356 animals were fed with either vector control, daf-2 RNAi, a 1∶1 mix of daf-2 and sir-2.4 RNAi, or a 1∶1 mix of daf-2 and yk615e RNAi bacteria from the time of hatching. yk615e is a gene randomly selected as a negative control in double RNAi experiments. Animals (n = 125 or greater) were scored for DAF-16::GFP nuclear translocation as described in Figure 4B. (PDF) [file pgen.1002948.s002.pdf]

Fig. S3

A.

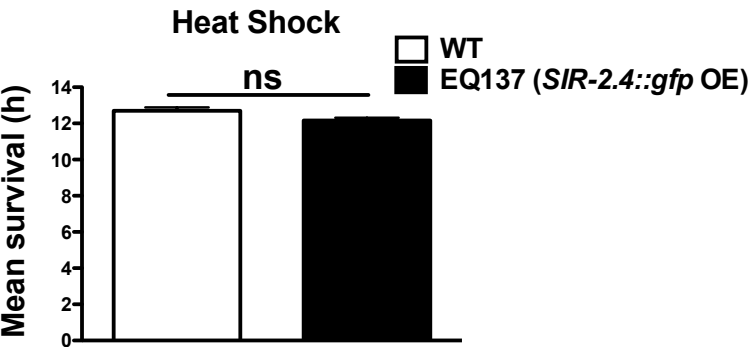

B.

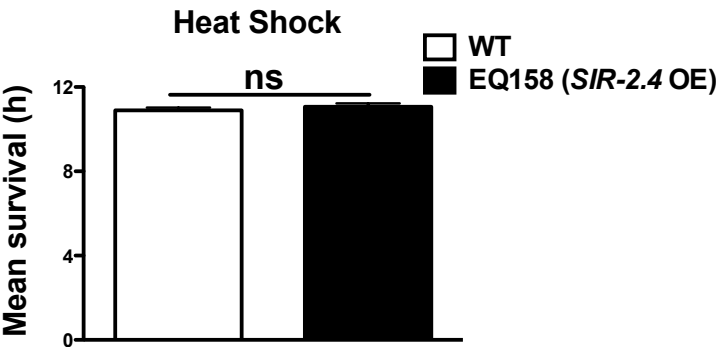

C.

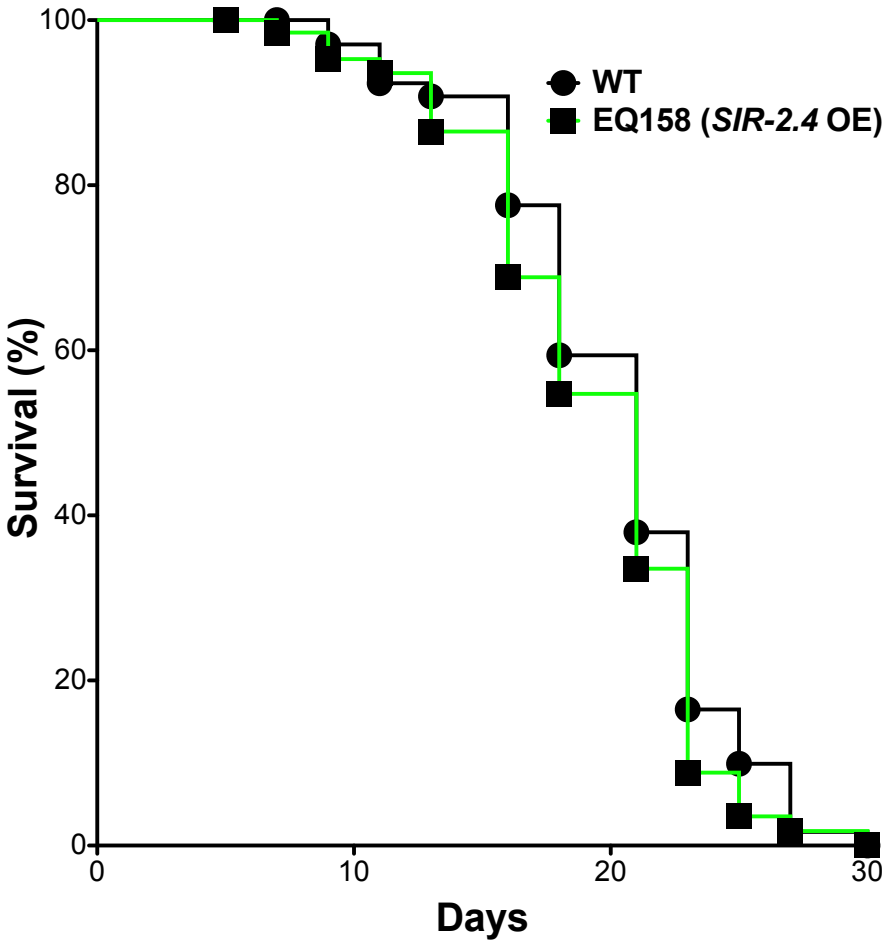

Supplement: Figure S3 — Minimal impact of SIR-2.4 overexpression on stress resistance or lifespan. Transgenic animals overexpressing (A) a SIR-2.4::GFP fusion protein or (B) native untagged SIR-2.4 protein and N2 controls were exposed to 35°C heat stress. Viability was then scored at the timepoints indicated. (C) Survival curves of wild-type (N2) animals or transgenic animals overexpressing native SIR-2.4 (EQ158) at 20°C. All statistical details are presented in Tables S1 and S2. (PDF) [file pgen.1002948.s003.pdf]

Fig. S4

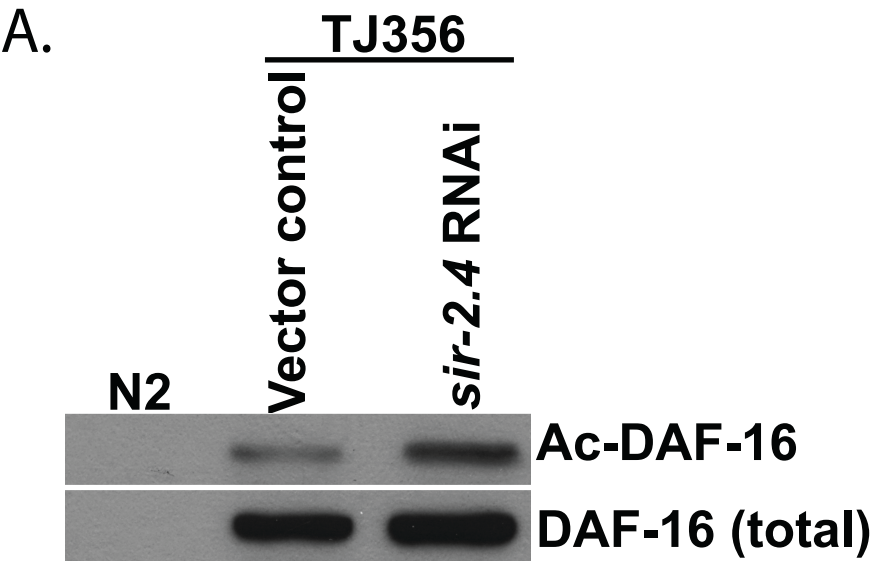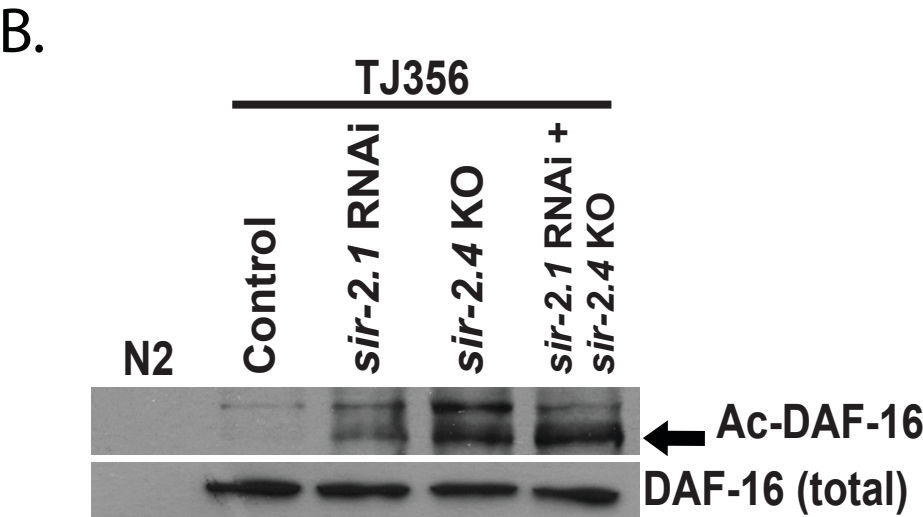

Supplement: Figure S4 — sir-2.4 RNAi promotes DAF-16 hyperacetylation. (A) DAF-16 acetylation was assessed in control or sir-2.4 RNAi worms by acetyl-lysine immunoprecipitation followed by GFP immunoblot as described in Figure 5A. (B) DAF-16 acetylation was assessed in sir-2.4 KO, sir-2.1 RNAi, or double loss-of function animals as indicated. (PDF) [file pgen.1002948.s004.pdf]

Fig. S5

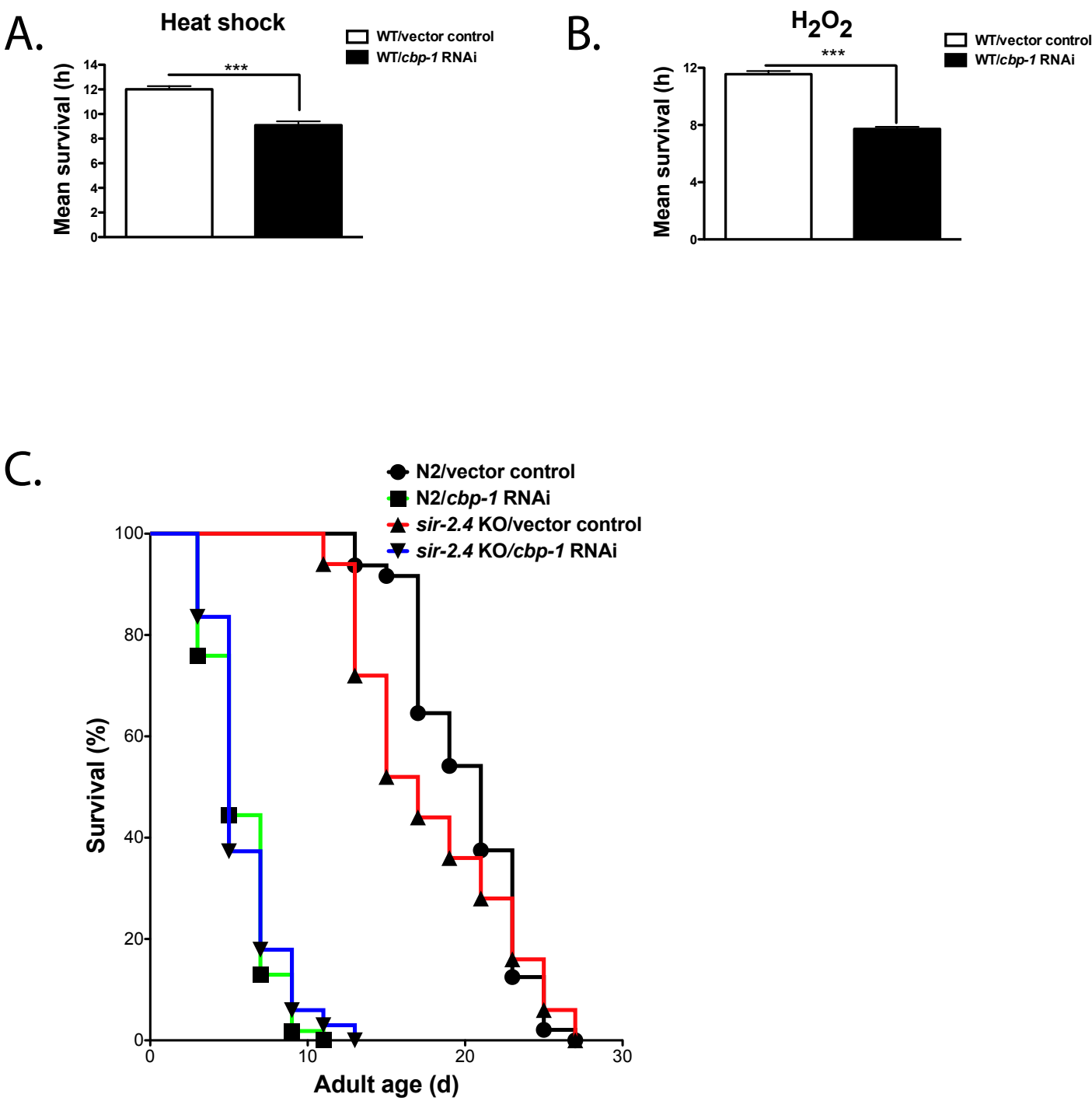

Supplement: Figure S5 — cbp-1 loss of function confers stress sensitivity and shortened lifespan. Mean survival of cbp-1 RNAi worms in response to heat shock (A) or peroxide stress (B). (C) Lifespan curves of wildtype or sir-2.4 KO animals in the presence of cbp-1 or control RNAi bacteria. (PDF) [file pgen.1002948.s005.pdf]
