# Supplementary material for: C. elegans SIRT6/7 Homolog SIR-2.4 Promotes DAF-16 Relocalization and Function during Stress
Source: PLoS Genet. 2012 Sep 13;8(9):e1002948. doi: 10.1371/journal.pgen.1002948 (PMC3441721; doi:10.1371/journal.pgen.1002948)
Supplement: Table S1 — Effects of sir-2.4 expression on stress resistance. Animals indicated were exposed to heat or oxidative stress. Mean survival ± SEM, in hours, observed in the stress analysis is shown. 75th percentile is the time at which the fraction of animals alive reaches 0.25. ‘n’ indicates the number of animals scored in the each experiment. P-Values calculated by pair-wise comparisons to vector control of the same experiment. We used Stata 8 software for statistical analysis and to determine means and percentiles. The logrank (Mantel-Cox) test was used to test the hypothesis that the survival functions among groups were equal. a P-Values calculated by pair-wise comparisons to N2 grown on vector control of the same experiment. b Compared to N2 grown on the same RNAi bacteria. c Compared to the same mutants grown on vector control. ‘*’ indicates the sets of experiments plotted and shown in Figures. (PDF) [file pgen.1002948.s006.pdf]

Table S1

Strains	Stress	Mean survival ± s.e.m. (hours)	75th percentile (hours)	n	*P*
**N2, control ***	**Heat-shock**	**12.05 ± 0.05**	**13**	**94/102**	
N2, daf-16(RNAi) *	**Heat-shock**	**9.89 ± 0.10**	**11**	**101/102**	**< 0.0001[a]**
***sir-2.4(n5137),* control ***	**Heat-shock**	**10.30 ± 0.11**	**11**	**96/102**	**< 0.0001[a]**
sir-2.4(n5137), daf-16(RNAi) *	**Heat-shock**	**9.63 ± 0.09**	**10**	**100/102**	**< 0.0001[a], 0.0611[b], 0.0001[c]**
**N2, control**	**Heat-shock**	**11.04 ± 0.08**	**12**	**102/120**	
N2, daf-16(RNAi)	**Heat-shock**	**9.46 ± 0.09**	**11**	**109/120**	**< 0.0001[a]**
***sir-2.4(n5137),* control**	**Heat-shock**	**10.68 ± 0.06**	**11**	**80/90**	**< 0.0228[a]**
sir-2.4(n5137), daf-16(RNAi)	**Heat-shock**	**9.81 ± 0.08**	**11**	**87/90**	**< 0.0001[a], 0.0936[b], 0.0001[c]**
**N2, control ***	H_2O_2	**10.31 ± 0.21**	**12**	**86/90**	
**N2, *sir-2.4(RNAi)* ***	H_2O_2	**7.30 ± 0.24**	**8**	**88/90**	**< 0.0001[a]**
***daf-16(mu86),* control ***	H_2O_2	**7.63 ± 0.21**	**10**	**89/90**	**< 0.0001[a]**
***daf-16(mu86), sir-2.4(RNAi)* ***	H_2O_2	**6.87 ± 0.12**	**8**	**90/90**	**< 0.0001[a], 0.0004[b], 0.2423[c]**
**N2, control**	H_2O_2	**10.96 ± 0.23**	**12**	**90/90**	
**N2, *sir-2.4(RNAi)***	H_2O_2	**8.95 ± 0.33**	**10**	**88/90**	**< 0.0001[a]**
***daf-16(mu86),* control**	H_2O_2	**7.98 ± 0.23**	**10**	**86/90**	**< 0.0001[b]**
daf-16(mu86), sir-2.4(RNAi)	H_2O_2	**8.59 ± 0.37**	**10**	**84/90**	**< 0.0001[a], 0.1728[b], 0.6965[c]**
**N2**	**Heat-shock**	**16.07 ± 0.20**	**18**	**79/84**	**-**
sir-2.4(n5137)	**Heat-shock**	**14.71 ± 0.17**	**16**	**79/84**	**< 0.0001**
**N2**	H_2O_2	**8.75 ± 0.09**	**8**	**72/72**	**-**
sir-2.4(n5137)	H_2O_2	**7.65 ± 0.11**	**8**	**72/72**	**< 0.0001**
**N2 ***	**Heat-shock**	**12.84 ± 0.61**	**19**	**98/140**	**-**
***daf-16(RNAi)* ***	**Heat-shock**	**9.81 ± 0.32**	**17**	**102/140**	**0.0001**
***sir-2.4(RNAi)* ***	**Heat-shock**	**9.34 ± 0.19**	**17**	**143/176**	**< 0.0001**
**N2**	**Heat-shock**	**15.52 ± 1.02**	**24**	**116/140**	**-**
daf-16(RNAi)	**Heat-shock**	**11.70 ± 0.32**	**20**	**134/140**	**< 0.0001**
sir-2.4(RNAi)	**Heat-shock**	**11.07 ± 0.19**	**20**	**163/176**	**< 0.0001**
**N2**	**Heat-shock**	**15.84 ± 0.74**	**22**	**103/160**	**-**
daf-16(RNAi)	**Heat-shock**	**11.70 ± 0.68**	**15**	**118/160**	**0.0004**
sir-2.4(RNAi)	**Heat-shock**	**10.53 ± 0.41**	**15**	**145/196**	**< 0.0001**
**N2 ***	H_2O_2	**7.58 ± 0.06**	**8**	**140/160**	**-**
***daf-16(RNAi)* ***	H_2O_2	**5.33 ± 0.08**	**5.5**	**156/160**	**< 0.0001**
***sir-2.4(RNAi)* ***	H_2O_2	**5.14 ± 0.08**	**5.5**	**191/196**	**< 0.0001**
**N2**	H_2O_2	**11.68 ± 0.10**	**8**	**134/160**	**-**
daf-16(RNAi)	H_2O_2	**5.81 ± 0.06**	**5**	**152/160**	**< 0.0001**
sir-2.4(RNAi)	H_2O_2	**5.68 ± 0.10**	**5**	**183/196**	**< 0.0001**
**N2; control**	**Heat-shock**	**12.01 ± 0.26**	**13**	**65/72**	**-**
**N2; *cbp-1(RNAi)***	**Heat-shock**	**9.09 ± 0.32**	**11**	**68/72**	**<0.0001**
**N2; control**	H_2O_2	**11.56 ± 0.22**	**13**	**72/72**	**-**
**N2; *cbp-1(RNAi)***	H_2O_2	**7.72 ± 0.16**	**9**	**72/72**	**<0.0001**
**N2 ***	**Heat-shock**	**17.08 ± 0.25**	**19**	**81/90**	**-**
**EQ137 (*sir-2.4::gfp* o.e.) ***	**Heat-shock**	**16.16 ± 0.24**	**19.2**	**86/86**	**0.1506**
**N2**	**Heat-shock**	**12.70 ± 0.18**	**13**	**65/65**	**-**
**EQ137 (*sir-2.4::gfp* o.e.)**	**Heat-shock**	**12.16 ± 0.16**	**14**	**54/72**	**0.4989**
**N2 ***	**Heat-shock**	**10.90 ± 0.13**	**11**	**36/36**	**-**
**EQ158 (*sir-2.4* o.e.) ***	**Heat-shock**	**11.07 ± 0.16**	**11.5**	**30/30**	**0.4654**

Table S1. Effects of sir-2.4 expression on stress resistance. Animals grown on vector control, daf-16 or sir-2.4 RNAi bacteria were exposed to heat or oxidative stress. Mean survival ± SEM, in hours, observed in the stress analysis was shown in the table. 75th percentile is the time at which the fraction of animals alive reaches 0.25. 'n' indicates the number of animals scored in the each experiment. *P*-Values calculated by pair-wise comparisons to vector control of the same experiment. We used Stata 8 software for statistical analysis and to determine means and percentiles. The logrank (Mantel-Cox) test was used to test the hypothesis that the survival functions among groups were equal. [a] *P*-Values calculated by pair-wise comparisons to N2 grown on vector control of the same experiment. [b] Compared to N2 grown on the same RNAi bacteria. [c] Compared to the same mutants grown on vector control. '*' indicates the sets of experiments plotted and shown in Figures.
