# Supplementary material for: C. elegans SIRT6/7 Homolog SIR-2.4 Promotes DAF-16 Relocalization and Function during Stress
Source: PLoS Genet. 2012 Sep 13;8(9):e1002948. doi: 10.1371/journal.pgen.1002948 (PMC3441721; doi:10.1371/journal.pgen.1002948)
Supplement: Table S2 — Effects of SIR-2.4 on lifespan. Adult mean lifespan ± SEM, in days, observed in lifespan analyses. Lifespan experiments were carried out at 20°C. 75th percentile is the age at which the fraction of animals alive reaches 0.25. ‘n’ shows the number of observed deaths relative to total number of animals started at day 1. The difference between these numbers represents the number of animals censored during the experiment, and includes animals that exploded, bagged (i.e. exhibited internal progeny hatching), or crawled off the plates. a p-Values calculated by pair-wise comparisons to N2 control of the same experiment. b p-Values calculated by pair-wise comparisons to mutants fed with control bacteria of the same experiment. We used Stata 8 software for statistical analysis and to determine means and percentiles. The logrank (Mantel-Cox) test was used to test the hypothesis that the survival functions among groups were equal. (PDF) [file pgen.1002948.s007.pdf]

Table S2

Strains	Mean lifespan ± s.e.m. (days)	75th percentile (days)	n	*P*
**N2; control**	**17.57 ± 0.24**	**24**	**57/60**	**-**
**N2; *sir-2.4(RNAi)***	**18.87 ± 0.55**	**24**	**73/75**	**0.0638**
***daf-2(e1370);* control**	**50.23 ± 0.91**	**63**	**64/90**	**< 0.0001**
daf-2(e1370); sir-2.4(RNAi)	**48.43 ± 0.61**	**60**	**68/90**	**< 0.0001[a]/ 0.5428[b]**
**N2; control**	**20.16 ± 1.03**	**23**	**48/59**	**-**
**N2; *cbp-1(RNAi)***	**5.79 ± 0.42**	**7**	**54/60**	**<0.0001**
***sir-2.4(n5137);* control**	**18.15 ± 1.15**	**23**	**50/71**	**0.1988**
sir-2.4(n5137); cbp-1(RNAi)	**6.16 ± 0.26**	**7**	**67/76**	**< 0.0001[a,b]/ 0.3274[c]**
**N2**	**16.51 ± 0.72**	**20**	**63/72**	**-**
**EQ137 (*sir-2.4::gfp* o.e.)**	**17.85 ± 0.66**	**22**	**53/72**	**0.1318**
**N2**	**20.18 ± 0.62**	**23**	**61/72**	**-**
**EQ158 (*sir-2.4* o.e.)**	**19.33 ± 0.63**	**23**	**57/72**	**0.2818**

Table S2. Effects of *sir-2.4* expression on lifespan. Adult mean lifespan ± SEM, in days, observed in lifespan analysis. Lifespan experiments were carried out at 20°C. 75th percentile is the age at which the fraction of animals alive reaches 0.25. 'n' shows the number of observed deaths relative to total number of animals started at day 1. The difference between these numbers represents the number of animals censored during the experiment, and includes animals that exploded, bagged (i.e. exhibited internal progeny hatching), or crawled off the plates. [a] *p*-Values calculated by pair-wise comparisons to N2 control of the same experiment. [b] *p*-Values calculated by pair-wise comparisons to mutants fed with control bacteria of the same experiment. [c] *p*-Values calculated by pair-wise comparisons to N2 fed with the same RNAi bacteria of the same experiment. We used Stata 8 software for statistical analysis and to determine means and percentiles. The logrank (Mantel-Cox) test was used to test the hypothesis that the survival functions among groups were equal.
