# Supplementary material for: C. elegans SIRT6/7 Homolog SIR-2.4 Promotes DAF-16 Relocalization and Function during Stress
Source: PLoS Genet. 2012 Sep 13;8(9):e1002948. doi: 10.1371/journal.pgen.1002948 (PMC3441721; doi:10.1371/journal.pgen.1002948)
Supplement: Table S3 — Peptides representing unique acetylated sequences from the FOXO transcription factor are shown with additional information. A“#” sign indicates the site of acetylation in the sequence. The site position refers to which lysine residue on FOXO the identified acetylation site represents. The mass to charge ratio (m/z) and charge state of the peptide as they were observed in the mass spectrometer are given. The mass error specifies the difference between the observed m/z ratio and the theoretical m/z ratio of a peptide, reported in part per million (PPM). The XCorr is the cross correlation score between the theoretical and observed MS2 spectra for the matched peptide. The unique ΔCorr is the difference between the XCorr of the top ranking peptide match (the reported peptide) and the XCorr of the next closest ranked peptide of a unique primary sequence (not simply an alternate placement of the acetylation site), normalized by the XCorr of the top ranking peptide match. All reported peptides passed a cutoff of a false discovery rate (FDR) <0.1%, based on the target-decoy strategy [61]. See Text S1 for materials and methods used in these studies. (DOCX) [file pgen.1002948.s008.docx]

| Peptide | Site Position | Observed m/z | Charge | Mass Error (PPM) | XCorr | Unique  ΔCorr |
| --- | --- | --- | --- | --- | --- | --- |
| SNTIETTTK#AQLEK#SR | K248, K253 | 630.997 | 3 | 1.25 | 5.094 | 0.440 |
| SNTIETTTK#AQLEK | K248 | 803.420 | 2 | 0.36 | 4.585 | 0.452 |
| IDATTHIGGVQIK#QESKPIK | K375 | 735.746 | 3 | 0.19 | 4.956 | 0.547 |
| QESK#PIKTEPIAPPPSYHELNSVR | K379 | 690.615 | 4 | 1.69 | 2.96 | 0.076 |

**Table S3:** Peptides representing unique acetylated sequences from the FOXO transcription factor are shown with additional information. A“#” sign indicates the site of acetylation in the sequence. The site position refers to which lysine residue on FOXO the identified acetylation site represents. The mass to charge ratio (m/z) and charge state of the peptide as they were observed in the mass spectrometer are given. The mass error specifies the difference between the observed m/z ratio and the theoretical m/z ratio of a peptide, reported in part per million (PPM). The XCorr is the cross correlation score between the theoretical and observed MS^2^ spectra for the matched peptide. The unique ΔCorr is the difference between the XCorr of the top ranking peptide match (the reported peptide) and the XCorr of the next closest ranked peptide of a unique primary sequence (not simply an alternate placement of the acetylation site), normalized by the XCorr of the top ranking peptide match. All reported peptides passed a cutoff of a false discovery rate (FDR) <0.1%, based on the target-decoy strategy [61].
