# Supplementary material for: C. elegans SIRT6/7 Homolog SIR-2.4 Promotes DAF-16 Relocalization and Function during Stress
Source: PLoS Genet. 2012 Sep 13;8(9):e1002948. doi: 10.1371/journal.pgen.1002948 (PMC3441721; doi:10.1371/journal.pgen.1002948)
Supplement: Text S1 — Supplemental materials and methods. (DOCX) [file pgen.1002948.s009.docx]

**Supplemental Materials and Methods**

**Sample Preparation/Digestion for LC-MS analysis**

A Coomassie-stained gel band corresponding to DAF-16 protein was excised, cut in to ~1 mm cubes and transferred to a 0.5 mL eppendorf tube. The gel pieces were destained using 50 % CH_3_CN/50 mM NH_4_HCO_3_. Gel pieces were dehydrated in 100% CH_3_CN. The dehydrated gel was rehydrated with a sufficient volume of 50 mM NH_4_HCO_3_ (containing 12.5 ng/µL trypsin) to cover all pieces (~20 µL). An additional volume of 50 mM NH_4_HCO_3_ (without enzyme) was added to the rehydrated pieces after an incubation period of 1 hr on ice, and the mixture was placed at 37° C overnight. The supernatant and two 20 µL extractions of the gel pieces using 50% CH_3_CN/5% HCOOH were collected in a 0.5 mL eppendorf tube and dried by vacuum centrifugation.

**LC-MS Analysis**

Dried peptides were resuspended in 5 µl of 5% CH_3_CN /4% HCOOH. 4 µL were loaded onto a pulled fused silica microcapillary column packed with 0.5 cm of Magic C4 resin (5 μm, 100 Å; Michrom Bioresources) followed by 20 cm of Maccel C18AQ resin (3 μm, 200 Å; Nest Group) using a Famos autosampler (LC Packings). Once loaded, the peptides were separated using an Accela 600 series quaternary pump (Thermo Scientific) across a 60 min linear gradient of 6% to 33% CH_3_CN in 0.125% HCOOH at a flow rate of ~300 nL/min. Peptides were detected in a hybrid dual-cell quadrupole linear ion trap–orbitrap mass spectrometer (LTQ Orbitrap Velos, Thermo Fisher) by means of a data-dependent Top20 method [[1](#_ENREF_64)].

**Database Searches and Data Filtering**

Raw files from the LC-MS analysis were first converted into mzXML format. All MS/MS spectra were searched using the SEQUEST algorithm (version 28) [[2](#_ENREF_65)]. Spectra were matched against a database consisting of sequences from all C. elegans open reading frame (ORFs) annotated in the Swiss-Prot database ([www.uniprot.org/uniprot/?query=taxonomy%3a6239&force=yes&format=fasta](http://www.uniprot.org/uniprot/?query=taxonomy%3a6239&force=yes&format=fasta), downloaded August 22^nd^, 2011). Each protein sequence was listed in both forward and reversed orientations to facilitate the estimation of the peptide identification false discovery rate (FDR). Data were searched using the following parameters: 50 ppm precursor mass tolerance and 1.0 Da product ion mass tolerance; up to two missed cleavages were allowed; A static modification of cysteine carbamidomethylation (+57.0214) was set. Variable modifications of methionine oxidation (+15.9949) and lysine acetylation (+42.0105) were also permitted.

The target-decoy method was employed to distinguish correct and incorrect identifications, in order to control the peptide FDR [[3](#_ENREF_66)]. Linear discriminant analysis using such parameters as XCorr, ΔCn, precursor mass error, number of missed cleavages and charge state was used to separate out correct and incorrect identifications [[4](#_ENREF_67)]. Peptides were order by descending discriminant score and filtered to a 1% FDR based on the number of decoy (reverse) sequences in the remaining data set.

**Supporting references**

1. Wu R, Dephoure N, Haas W, Huttlin EL, Zhai B, et al. (2011) Correct interpretation of comprehensive phosphorylation dynamics requires normalization by protein expression changes. Mol Cell Proteomics 10: M111 009654.

2. Eng J, McCormack AL, Yates JR (1994) An approach to correlate tandem mass spectral data of peptides with amino acid sequences in a protein database. J Am Soc Mass Spectrometry 5: 976-989.

3. Elias JE, Gygi SP (2007) Target-decoy search strategy for increased confidence in large-scale protein identifications by mass spectrometry. Nat Methods 4: 207-214.

4. Huttlin EL, Jedrychowski MP, Elias JE, Goswami T, Rad R, et al. (2010) A tissue-specific atlas of mouse protein phosphorylation and expression. Cell 143: 1174-1189.
